# A Stemness and EMT Based Gene Expression Signature Identifies Phenotypic Plasticity and is A Predictive but Not Prognostic Biomarker for Breast Cancer

**DOI:** 10.7150/jca.34649

**Published:** 2020-01-01

**Authors:** Muhammad Waqas Akbar, Murat Isbilen, Nevin Belder, Secil Demirkol Canli, Baris Kucukkaraduman, Can Turk, Ozgur Sahin, Ali Osmay Gure

**Affiliations:** 1 Department of Molecular Biology and Genetics, Bilkent University, Ankara, Turkey.; 2 DNAFect Genetics Consulting R&D and Biotechnology Inc., Kocaeli, Turkey.; 3 Molecular Pathology Application and Research Center, Hacettepe University, Ankara, Turkey.

**Keywords:** Breast cancer, predictive biomarkers, tumor plasticity, transcriptomics.

## Abstract

**Aims:** Molecular heterogeneity of breast cancer results in variation in morphology, metastatic potential and response to therapy. We previously showed that breast cancer cell line sub-groups obtained by a clustering approach using highly variable genes overlapped almost completely with sub-groups generated by a drug cytotoxicity-profile based approach. Two distinct cell populations thus identified were CSC(cancer stem cell)-like and non-CSC-like. In this study we asked whether an mRNA based gene signature identifying these two cell types would explain variation in stemness, EMT, drug sensitivity, and prognosis *in silico* and *in vitro*. **Main methods:** *In silico* analyses were performed using publicly available cell line and patient tumor datasets. *In vitro* analyses of phenotypic plasticity and drug responsiveness were obtained using human breast cancer cell lines. **Key findings:** We find a novel gene list (CNCL) that can generate both categorical and continuous variables corresponding to the stemness/EMT (epithelial to mesenchymal transition) state of tumors. We are presenting a novel robust gene signature that unites previous observations related either to EMT or stemness in breast cancer. We show *in silico*, that this signature perfectly predicts behavior of tumor cells tested *in vitro*, and can reflect tumor plasticity*.* We thus demonstrate for the first time, that breast cancer subtypes are sensitive to either Lapatinib or Midostaurin. The same gene list is not capable of predicting prognosis in most cohorts, except for one that includes patients receiving neo-adjuvant taxene therapy. **Significance:** CNCL is a robust gene list that can identify both stemness and the EMT state of cell lines and tumors. It can be used to trace tumor cells during the course of phenotypic changes they undergo, that result in altered responses to therapeutic agents. The fact that such a list cannot be used to identify prognosis in most patient cohorts suggests that presence of factors other than stemness and EMT affect mortality*.*

## Introduction

Breast cancer (BC) is the second leading cause of mortality after lung cancer in women^1^. Currently the molecular classification of BC is based on expression of Estrogen Receptor (ER), Progesterone Receptor (PR) and Erb-B2 Receptor Tyrosine Kinase 2 (ERBB2). Another classification, which is also based on gene expression differences identifies luminal A, luminal B, Her2 enriched, basal, and normal like subgroups, and provides a better prediction of prognosis and drug response^2^,^3^. Some studies identified a cancer stem cell (CSC)-like subpopulation in BC, which was suggested as being responsible for metastasis and relapse of disease^4^,^5^. In general, CSCs are generally defined as CD44^+^/CD24^-^ cells that possess the capacity of self-renewal^6^. The CSC hypothesis suggests - by definition - that non-CSC cells would be generated from CSC cells. On the other hand, a related but distinct mechanism, i.e. epithelial to mesenchymal transition (EMT), describes a reversible process through which epithelial cells transform into a mesenchymal state with the associated loss of epithelial features such as cell to cell contact and intracellular tight junctions, and the gain of mesenchymal features such as fibroblastoid morphology, increased motility and metastatic capability^7^. Cells which undergo EMT show similar features to CSCs such as tumorigenesis, lack of differentiation, mammosphere formation and resistance to anti-cancer therapies, suggesting that these two might be defining the same phenotype^8^. Mesenchymal cells can switch to an epithelial state (MET) under certain conditions^9^,^10^. The presence of CSCs has been conclusively shown *in vivo*^11^ and *in vitro*^12^,^13^. It is known that CSC-like cells can be generated or enriched via mammosphere formation^14^ or the ectopic expression of Twist or Snail^15^. However, both of these are also known to induce EMT. It is not yet clear if cells defined as CSCs in BC are identical to mesenchymal cells, and whether if via EMT, epithelial cell populations in BC can revert back to a CSC-like state.

An important distinction between CSC-like and non-CSC-like cells (or epithelial and mesenchymal cells) is their differential response to anti-cancer therapy. Commonly used drugs like Paclitaxel and Doxorubicin have been shown to affect primarily non-CSC cells, thereby enriching CSC-like populations in tumors^16^,^17^. Therefore, for successful tumor therapy, it seems drugs which specifically target CSC-like cells are also required. Ultimately, by the use of two drugs alternately or in parallel, both CSC- and non-CSC like cells could be eliminated.

In this study, using cells classified according to a novel gene-list (CNCL), we were able to identify candidate drugs for both CSC- and non-CSC cell types in breast cancer and validated our findings in several datasets *in silico,* as well as* in vitro*. However, we were not able to observe a consistent difference in clinical outcome in the majority of BC patient cohorts whose tumors had been thus classified.

## Methods

### *In silico* analyses

For *in silico* gene expression analysis, microarray datasets were downloaded from genomic data hosting websites, ArrayExpress (https://www.ebi.ac.uk/arrayexpress/) and Gene expression Omnibus (GEO, http://www.ncbi.nlm.nih.gov/geo/). Each dataset was RMA normalized using BRB array tools^18^. FPKM values were used from GSE73526 which is a next generation RNA-sequencing dataset. Cluster tree 3.0 program^19^ was used to hierarchically cluster data and heatmaps were generated using Java Treeview^20^. For both genes and samples, Euclidean distances were calculated using complete linkage. Datasets generated using Affymetrix U133 A, B or U133 plus2 or Illumina platforms were used. Details of the datasets used are given in **Supplementary Table 1**. For *in silico* drug cytotoxicity analysis, IC50 or normalized activity area (AA) values were downloaded from CCLE^21^ and CGP^22^. We did not select patients in any of the cohorts utilizing any filters as we intended to test if CNCL could identify prognosis, independent of clinical characteristics.

### Cell culture conditions

Breast cancer cell lines, MDA-MB-157, MDA-MB-231, MDA-MB-453, MDA-MB-468, MDA-MB-436, ZR751, JIMT1, BT474, BT20, CAL51 and MCF7 were cultured in DMEM media, while HCC202, HCC1954, HCC70, HCC1143, HCC38, T47D and HCC1937 cell lines were cultured in RPMI media. Respective media was supplemented with 10% FBS, 1% 200mM L-Glutamine (Lonza) and 1% 10K/10K Penicillin -Streptomycin (Lonza) of the total volume. All cell lines were incubated at 5% CO_2_ and 37 ^0^C in humidified incubator.

### Mammosphere culture (3D culture)

MDA-MB-157 cells were cultured in 75cm^2^ low attachment flasks (Corning) to generate mammospheres (3D culture) in 3 separate experiments according to a previously published protocol^23^. Briefly, to generate mammosphere media, serum free DMEM media was enriched with 1X B27 (Invitrogen), 10 ng/ml EGF (Sigma Aldrich), FGF 20 ng/ml (Sigma Aldrich), 2 µg/ml heparin (Sigma Aldrich), L-Glutamine (Lonza) and Penicillin -Streptomycin (Lonza). To initiate mammosphere cultures, cells grown in monolayers were detached and resuspended in mammosphere media. Cells were counted and 2x10^5^ cells were cultured in 75cm^2^ low attachment flasks. After 3 days of culture, cells formed mammospheres. These mammospheres were separated from suspension using a 40 µm cell strainer (BD). Spheres were retrieved from the strainer by inverting it over petri dish and washing with PBS. After which cells were replated in low attachment flasks. This was carried out for 6 passages.

### Quantitative RT-PCR

Total RNA was isolated using Trizol (Ambion) and was treated with DNAse (Ambion) according to manufacturer's protocols. RNA was reverse transcribed by Revert-Aid first strand cDNA synthesis kit (Thermo Fisher Scientific) according to the supplier's protocol using random hexamer primers. Quantitative profiling of selected genes by qRT-PCR was performed in triplicates using Light Cycler 480 (Roche) with iTaq Universal SYBR Green Supermix (Bio-Rad). Primers were designed using Primer3 online tool (http://primer3.ut.ee/) and then validated with NCBI Primer-Blast Tool (http://www.ncbi.nlm.nih.gov/tools/primer-blast/). Primer sequences are shown in **Supplementary Table 2.** GAPDH was used as endogenous reference control. All expression data were calculated using the ΔΔCT Method^24^.

### Cell viability analyses

Cell viability was analyzed in quadruplicates using CellTiter-Glo® Luminescent Cell Viability Assay Kit (Promega) and CyQUANT Cell Proliferation Assay Kit (Thermo Fisher Scientific) according to manufacturer's instructions. For drug cytotoxicity in 2D culture, 3000 cells were plated in each well of 96 well plates and after 24 hours were treated with drugs (Lapatinib, Midostaurin) at 10 different doses (Lapatinib: 50 µM-0.001 µM, Midostaurin: 5µM-0.0001 µM). Percent cell viability was calculated 72 hours after drug treatment. These values were then used to draw dose-inhibition curves using 6 models as (3 parameter, 3 parameter Top 100, 3 parameter Bottom 0, 4 parameter, 4 parameter Top 100 and 4 parameter Bottom 0) using R software. The model with the lowest standard error was used to calculate IC50 and Activity area values. Same procedures were used to calculate cytotoxicity for mammosphere cultures except mammosphere generated cells were cultured in 96 well ultra-low attachment plates in mammosphere media.

### siRNA knockdown and cytotoxicity change

Knockdown of ZEB1 and SNAI2 genes was performed using commercially available siRNAs, targeting the respective transcripts (D-006564-04 (SiZEB1) and D-017386-02 (SNAI2), Dharmacon Lafayette, CO, USA) along with control siRNA from Qiagen (CAT# SI03650318, Qiagen, Hilden, Germany) using Lipofectamine 2000 (Invitrogen, Carlsbad, CA ) in serum free Opti-MEM at a concentration of 20 nM according to the manufacturer's protocol. In brief, cells were seeded at a density of 2x10^5^ cells/well in complete medium. When the cells reached 50-60 % confluency, medium was replaced with medium without antibiotics. The siRNA-Lipofectamine 2000™ mixture was added to the medium and cells were incubated at 37°C and 5% CO2 for 48 hours. Cells were collected 72 h after transfection to analyze changes in transcript levels of related genes using QRT-PCR. For cytotoxicity experiments with Lapatinib and Midostaurin MDA-MB-157 cells (3000 cells/well) were seeded in 96-well plates and twenty-four hours after seeding, cells were transfected with the siZEB1 and siSNAI2 at a final concentration of 20 nM, using Lipofectamine 2000TM (Invitrogen, CA) transfection reagent according to the manufacturer's protocol. After 24 hours of transfection, cells were treated with at 4 concentrations for Lapatinib (1, 5, 10 and 50 µM) and Midostaurin (0.1, 0.5, 1 and 5 µM). Cell viability was assessed 72 hours after treatment with CellTiter-Glo Luminescent Cell Viability Assay (Promega, Madison, WI, USA) as per the manufacturer's instructions. All treatment groups were set up in quadruplicate.

### Statistical analysis

Different treatment groups were compared using “t-test” and graphs were generated using GraphPad Prism v 6.01 (GraphPad Software Inc.). For survival analysis, Cox proportional hazard regression and Log-Rank tests were performed using the “Survival” package in R^25^,^26^. Kaplan Meier analysis was performed using IBM SPSS Statistics v.19.

## Results

### Identification of the CSC/non-CSC gene list (CNCL)

**Figure 1:**
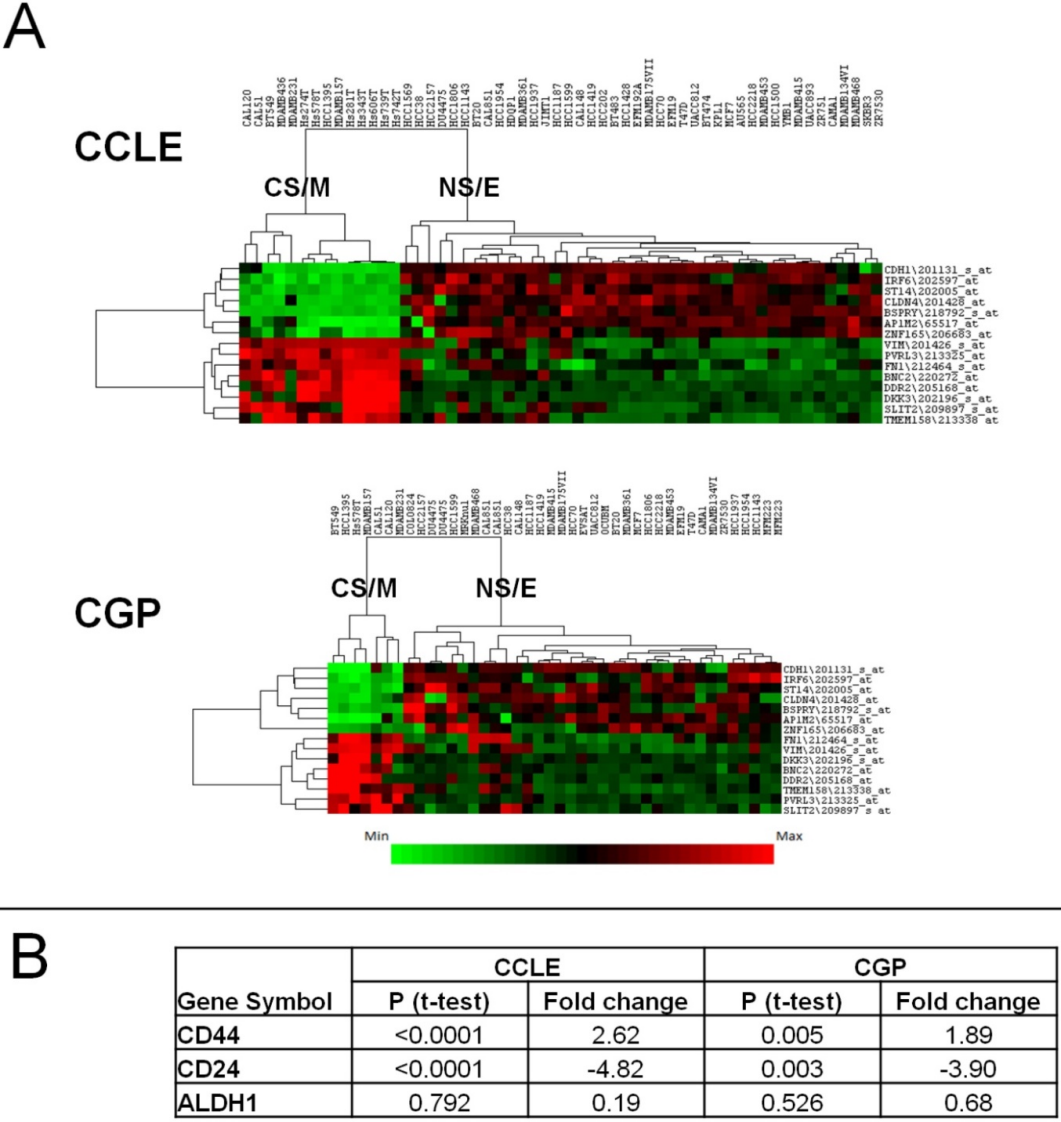
** Clustering of cell lines in CCLE and CGP datasets by CNCL.** CNCL generates two distinct clusters for CCLE and CGP cell lines** (A)**. CS/M and NS/E classification correlates with stem cell markers CD44 and CD24 but not with ALDH in both datasets (see also **Supplementary Table 6**). CD44 and CD24 expression is significantly different between CSC-like and non-CSC-like clusters by t-test but ALDH did not show the same pattern in CCLE and CGP datasets **(B).**

We previously showed^27^ that breast cancer cell line sub-groups obtained by a clustering approach using highly variable gene expression differences^28^ overlapped with that generated by a cytotoxicity based approach^16^. Thus, we could classify all breast cancer cell lines either as CSC-like or non-CSC-like. To generate a robust gene list that could be used to separate these two groups *in silico* and *in vitro*, we identified the most differentially expressed (p<0.05, by t-test) 200 genes among the CSC-like or non-CSC-like cells using both the GSE36139 (CCLE)^21^ and E-MTAB-783 (CGP)^22^ datasets, which contain 56 and 39 breast cancer cell lines, respectively. One hundred and thirty three probesets, corresponding to 97 genes were common to both datasets, and 129 had highly correlated expression patterns (**Supplementary Figures 1A and 1B**). Of these, we selected 15 genes/probesets (8 up and 7 down-regulated in CSC-like cell lines), which had a minimum fold-change of 3 and a p value below 0.0002 and showed strong intergenic correlation (**Supplementary Tables 3A and 3B**); thus forming the CSC/non-CSC gene list (CNCL). A hierarchical clustering analysis using CNCL shows two clearly distinct cell clusters in CCLE and CGP datasets (**Figure 1A**). These two clusters were designated cancer-stem-cell-like and mesenchymal (CS/M) and non-cancer-stem-cell-like and epithelial (NS/E) as explained below.

We then used these cell clusters to perform gene-set-enrichment analyses (GSEA)^29^. Genesets enriched in both CSC-like and non-CSC-like groups were ranked by significance, and rank numbers obtained in both analyses were combined generating a ranksum value to identify commonly enriched genesets. As shown in **Supplementary Tables 4A and 4B**, gene sets indicative of a mesenchymal phenotype such as “Extracellular Matrix”, “Basement Membrane” and “Cell Migration” were enriched in CSC-like cells, while gene sets related to an epithelial phenotype like “Tight Junction” were enriched in non-CSC-like cell lines, suggesting that the CSC-like phenotype is similar to a “mesenchymal”, and non-CSC-like to an “epithelial” phenotype. In support of this, an *in silico* analysis of CDH1 protein expression from two independent sources^30^,^31^ showed that CDH1 was significantly up-regulated among non-CSC-like cell lines compared to those with a CSC-like phenotype (**Supplementary Figure 2**). We thus conclude that CNCL classifies cell lines into relatively epithelial and mesenchymal phenotypes and therefore name these two types of cells CS/M and NS/E.

To test the robustness of the CNCL gene list we asked if cell lines defined as CS/M or NS/E in CGP and CCLE would be classified consistently by CNCL in other datasets as well. Cell line gene expression data from 3 datasets: GSE24717^32^ and GSE50811^33^ (microarray) and GSE73526^31^ (RNA sequencing) was used for this purpose. Of 123 cell line assignments, only 1 (HCC1419 in GSE50811) was classified inconsistently across datasets. We therefore, conclude that CNCL behaves consistently across different platforms and is unaffected from inter-laboratory variation (**Supplementary Table 5**).

We then asked if the CNCL gene list could provide biologically significant distinctions among cell and tissue subtypes* in silico* and* in vitro*.

### CNCL reflects stemmness *in silico*

**Figure 2:**
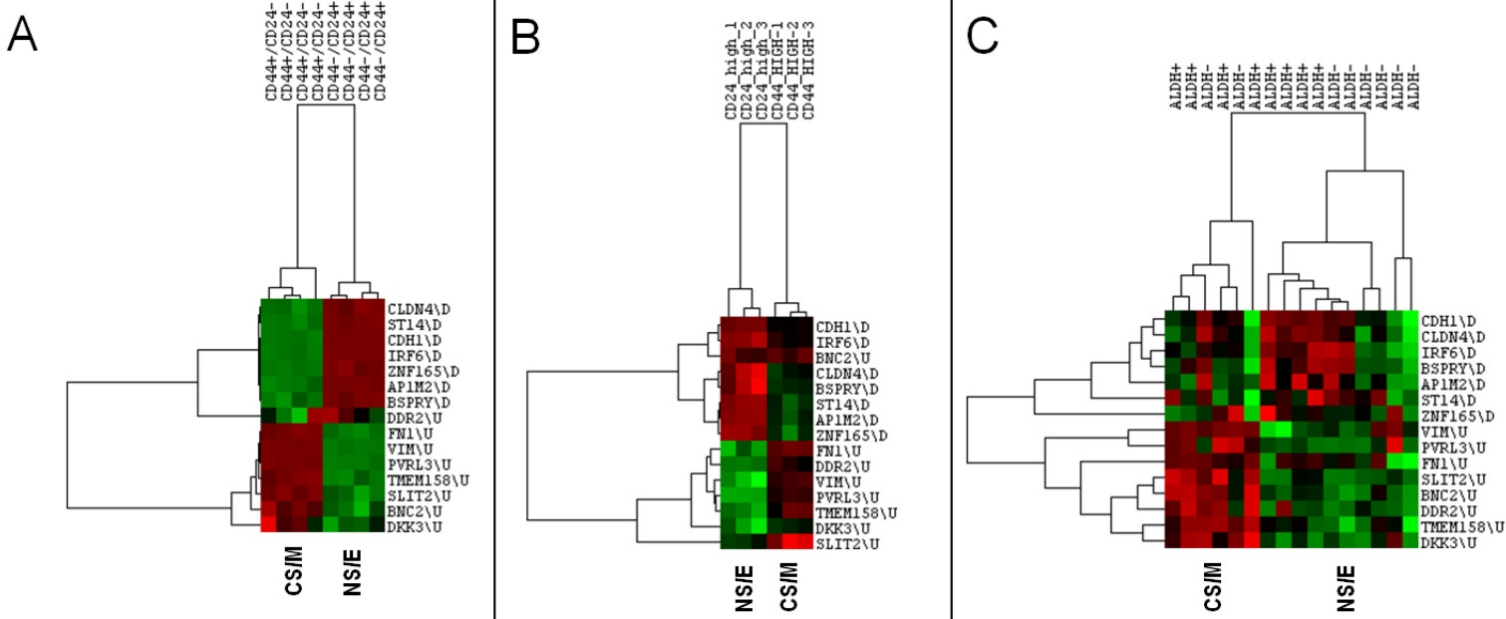
** CNCL identifies stemness *in silico*.** CNCL generates two clusters of CD44+/CD24- and CD44-/CD24+ MCF10A breast cancer cells (GSE15192) **(A),** as well as in Ras-transformed human mammary epithelial cells sorted based on their CD44 or CD24 expression (GSE36643) **(B).** In primary breast cancer cells sorted for ALDH+ expression (GSE52327), CNCL based differentiation is not clear **(C)**.

NS/E and CS/M cells identified by CNCL showed significantly different levels of expression, *in silico* for CD24 and CD44 in both CCLE and CGP datasets, while another stem-cell marker, ALDH did not show differential expression among cell groups (**Figure 1B**, and **Supplementary Table 6**). CNCL could clearly identify CD44+/CD24- stem cells and CD44-/CD24+ non stem cells derived from MCF10A breast cancer cells (GSE15192)^34^ (**Figure 2A**). Similarly, CNCL could distinguish stem and non-stem HMLER cells (Ras-transformed human mammary epithelial cells) obtained by CD44/CD24 expression sorting (GSE36643)^35^ (**Figure 2B**). The distinction between CS/M and NS/E type cells in primary breast cancer cells (GSE52327)^36^ was less clear (**Figure 2C**) possibly reflecting the heterogeneity of these cells. ALDH expression was not related to CNCL defined phenotypes in this analysis as well. Expression of all 15 genes in CNCL correlated with CD24 and CD44 but not with ALDH in GSE15192 **(Supplementary Table 7A)** and in GSE36643 **(Supplementary Table 7B)**, similar to CCLE or CGP. Cumulatively, these data suggest NS/E cells are highly similar non-stem cancer cells, while the opposite is true for CS/M cells.

### CNCL can identify EMT *in silico*

**Figure 3:**
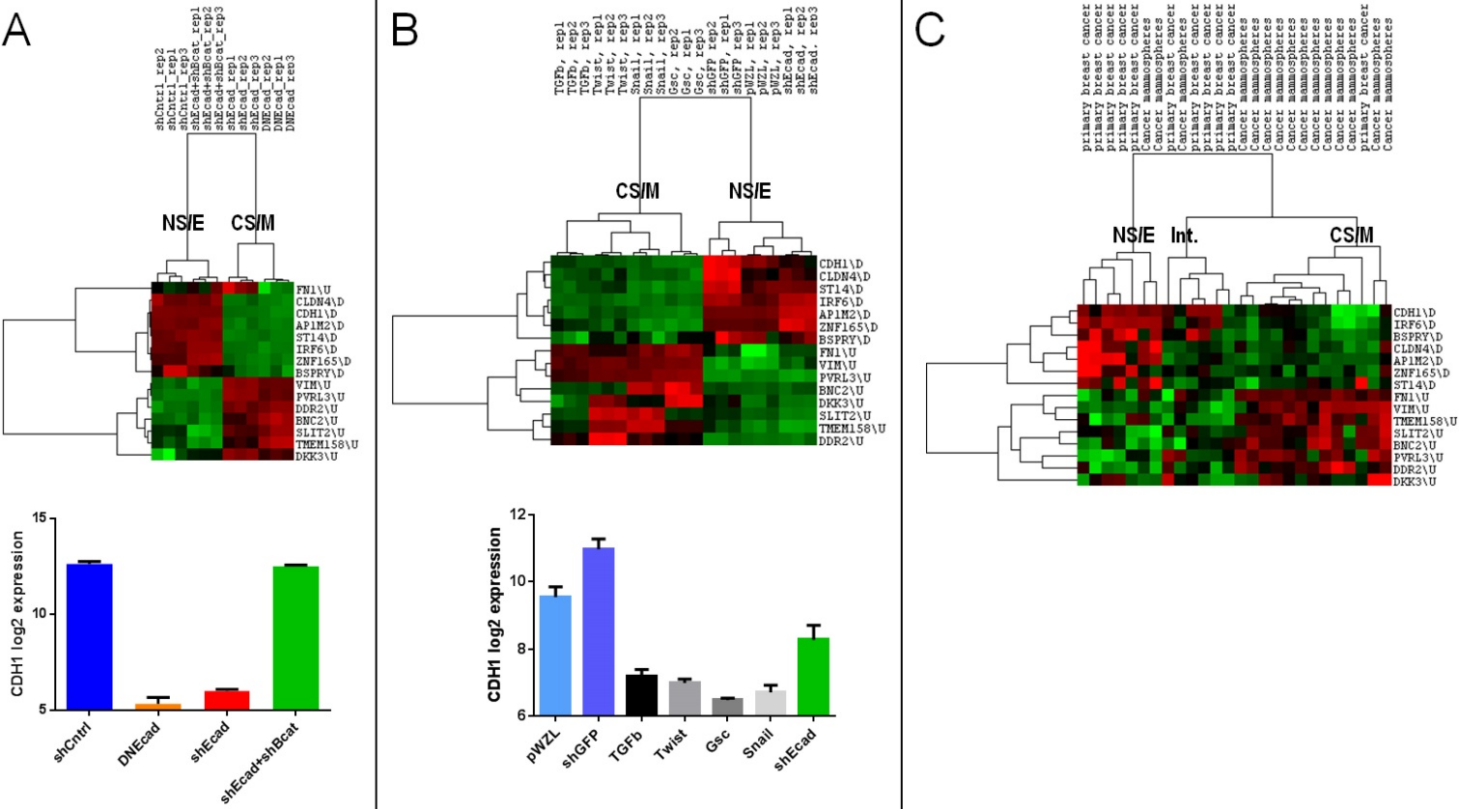
** CNCL identifies EMT *in silico*.** CNCL identifies cells in which CDH1 was downregulated using either shRNA (shEcad) or via dominant-negative CDH1 expression (DNEcad), as CS/M cells, when compared to controls (shCntrl) or cells where both CDH1 and CTNNB1 were downregulated (shEcad+shBcat) which are of the NS/E phenotype (GSE9691). CDH1 expression was significantly lower in CS/M as compared to NS/E cells (CDH1 vs. shCntrl: <0.0001, CDH1 vs. shCDH1+shBcat (CTNNB1) <0.0001) (**A**). In GSE24202, cells over-expressing TGF-β, Twist, Gsc or Snail are of the CS/M phenotype compared to controls (shGFP and pWZL). Although cells treated with shCDH1 downregulated CDH1, its expression remained higher than TGF-β (p=0.02), Twist (p<0.01), Gsc (p<0.01), and Snail (p<0.01) (**B**). For primary breast cancer cells and mammospheres generated from those samples CNCL identified most of the mammospheres (12/15) as CS/M and all but one primary cancer samples as NS/E and intermediary, in GSE7515 (**C**).

We next analyzed the relation of CNCL determined clusters with those determined in the context of EMT. When the GSE9691 dataset that contains expression data from HMLER cells in which E-Cad gene was downregulated either by shRNA treatment or by the transfection of a dominant negative version of E-Cad, or cells in which both E-Cad and β-Cat were downregulated^37^ were analyzed, CNCL correctly classified all E-Cad down-regulated cells as CS/M, and all control cells, and those where effects of E-Cad downregulation are reversed by simultaneous β-Cat downregulation, as NS/E cells (**Figure 3A**). In a separate analysis, HMLER cells that were retrovirally transduced with TGFβ, Twist, Gsc or Snail inducing EMT^38^, were identified by CNCL as CS/M cells (GSE24202) (**Figure 3B**). Unexpectedly when E-cad expression was downregulated by shRNA in these cells, they were still identified as NS/E. However, shRNA dependent downregulation of E-cad in these cells was significantly less efficient compared to the other experiments (TGFB, Twist, Snail and Gsc), suggesting that a conversion to a more mesenchymal state possibly requires E-cad downregulation below a certain threshold (**Figure 3B**, **bottom**). To test if CNCL could classify primary tumor cells we analyzed the GSE7515 dataset^39^. Of 11 primary tumor cells, 10 were classified either as NS/E or intermediary (I) phenotypes by CNCL, while 1 was of a CS/M phenotype. In contrast, among 15 mammospheres, generated from primary tumor cells, in which EMT would be expected to occur, 12 were characterized as CS/M, and 3 as either NS/E or I cells (**Figure 3C**). Therefore, 22 of 26 primary cell and mammosphere cultures were clustered by the CNCL list in an expected fashion. Exceptions could be due to the fact that primary cells can have a mesenchymal phenotype to start with, and because *in vitro* mammosphere cultures might not always generate mesenchymal cells. Cumulatively these results indicate that CNCL is able to distinguish cells with relatively epithelial features from those with mesenchymal features for both cell lines and primary tumor cells.

### CNCL can predict drug sensitivity *in silico*

**Figure 4:**
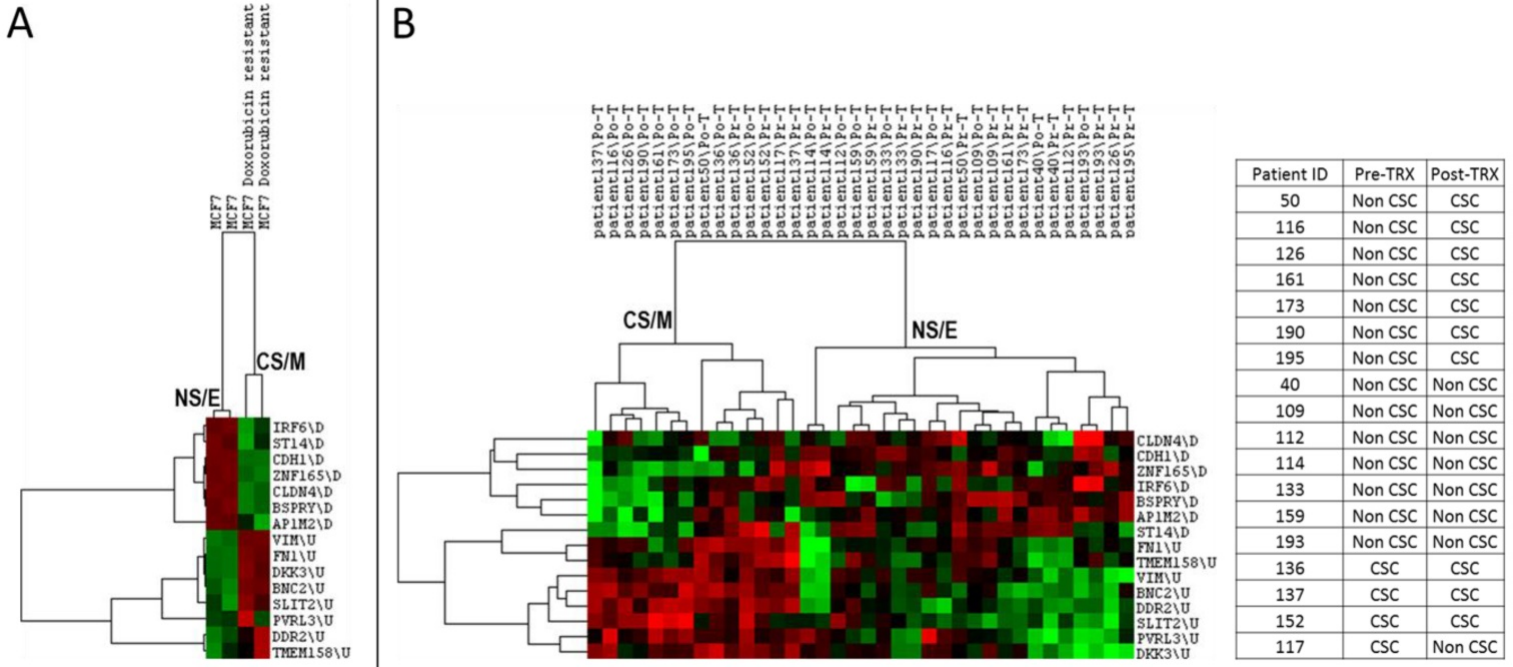
** CNCL can predict drug sensitivity *in silico*.** CNCL identifies doxorubicin resistant MCF7 cells as CS/M, compared to control cells (GSE24460) which are NS/E (**A**). (**B**): CNCL identifies Letrezol resistance in pre- and post- Letrezol treated tumor tissue (GSE10281) from 14 NS/E tumors, 7 converted to a CS/M phenotype, post treatment.

As both cancer stem cells and mesenchymal cancer cells have been reported to resist the majority of cancer chemotherapy and/or targeted therapy regimens, we asked if CNCL could identify drug sensitive and resistant cells *in silico*. As shown in **Figure 4A**, CNCL could clearly distinguish Doxorubicin sensitive MCF7 cells from those that were made resistant to this drug via prolonged exposure (GSE24460)^17^. Development of drug resistance *in vivo* is a major cause of therapy failure. To investigate if the CNCL list could identify a phenotype switch in *in vivo* drug resistance, we utilized the GSE10281 dataset which contains pre- and post- Letrezol treated tumor tissue from 18 patients^39^. CNCL based analysis of this dataset revealed that after a 3 month treatment period, out of 14 tumors with a pre-treatment NS/E phenotype, 7 had converted to CS/M tumors post-therapy. And among 4 tumors with a CS/M phenotype, pre-therapy, one converted to a NS/E tumor post-therapy (**Figure 4B**). We interpret these results to mean that most tumors with a NS/E phenotype pre-therapy, are prone to generate CS/M tumors post-therapy, and thus possibly become drug resistant.

### Analyses based on a quantitative stemness score (SS)

**Figure 5:**
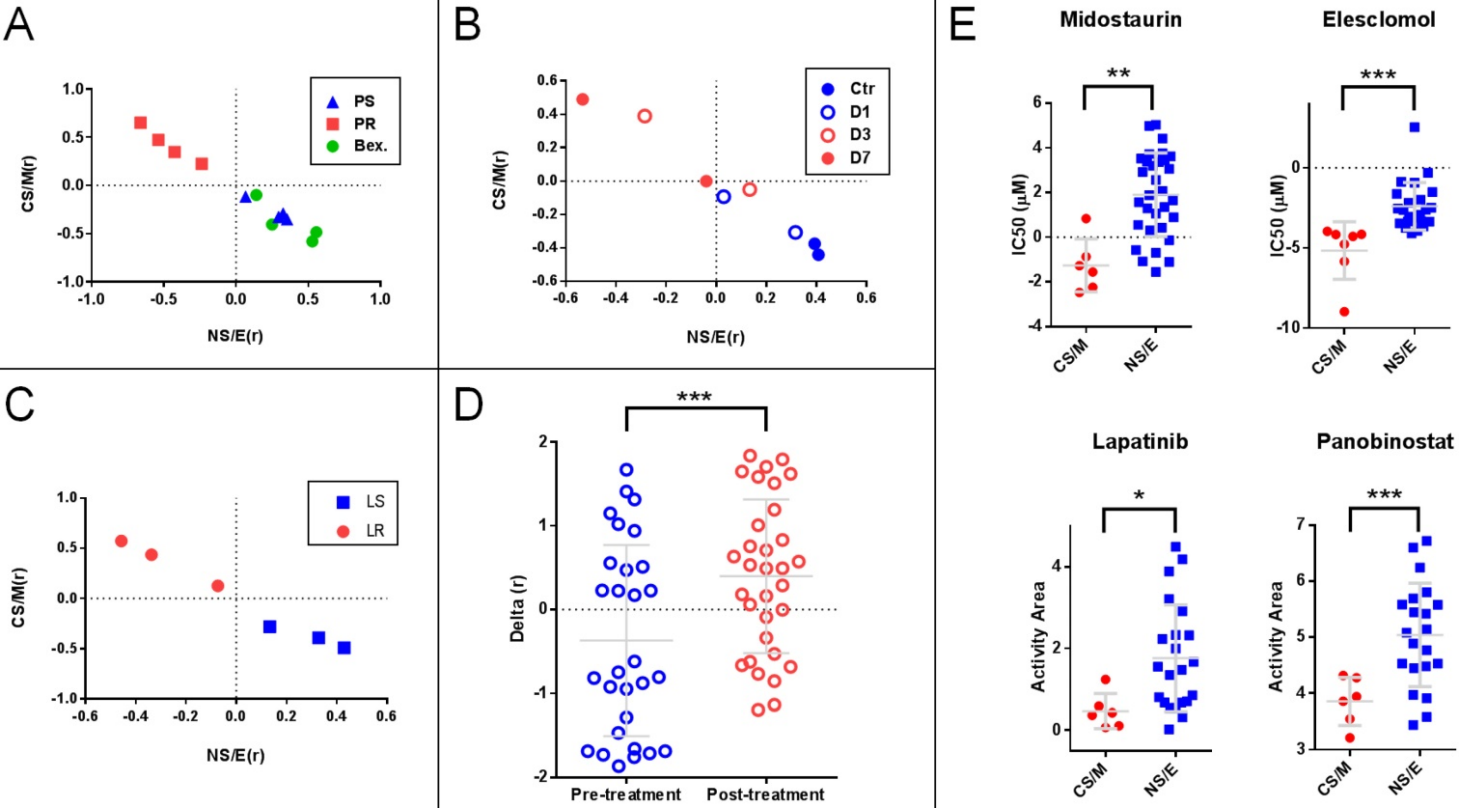
** Stemness score (SS) based analyses show that the CNCL can reflect tumor plasticity. A:** (GSE12791) Paclitaxel resistant MDA-MB-231 cells (PR) show high CS/M (r) and low NS/E (r) as compared to Paclitaxel sensitive cells (PS) or those PR cells treated with Bexarotene (Bex). **B:** (GSE23399) Paclitaxel treated carcinoma associated fibroblasts (CAFs) treated for 3 and 7 days (D3 and D7) show higher CS/M (r) and low NS/E (r) when compared to cells treated for 1 days or control cells (D1 and Ctr). **C:** (GSE16179) Lapatinib-resistant BT474 cell line (LR) show high CS/M (r) and low NS/E (r) when compared to sensitive cells (LS). **D:** (GSE28844) Patient tumors post-anthracycline and taxane chemotherapy, show high Delta (r) when compared to pre-treatment where Delta (r): [CS/M(r)] - [NS/E(r)]. **E:** Midostaurin and Elesclomol are cytotoxic preferentially for CS/M while Lapatinib and Panabinostat target NS/E. *p<0.05 (t-test), **p<0.01, ***p<0.001.

We generated two matrices, one for CS/M related-, and one for NS/E related genes (**Supplementary Table 8**) based on the median expression levels for each gene in the CNCL for CS/M and also for NS/E cells in the CCLE database after mean standardization. We then correlated each tumor sample's standardized CNCL gene expression values to both the CS/M and NS/E matrices generating two correlation values (Pearson's r) for each matrix, CS/M(r) and NS/E(r), respectively. Analysis of cells based on their correlation scores clearly distinguished Paclitaxel sensitive and resistant MDA-MB-231 cells in GSE12791, as well as cells re-sensitized to Paclitaxel through differentiation inducing Bexarotene treatment^40^ (**Figure 5A**). In another case (GSE23399), we found that the same analysis was able to reveal the phenotypic change observed in cancer-associated fibroblasts (CAF) in response to paclitaxel therapy^41^. As shown in **Figures 5B**, the longer CAFs were exposed to Paclitaxel, the more CS/M-like, and less NS/E-like they became. A similar switch to a more CS/M-like phenotype was also observed in BT20 breast cancer cells made resistant to a targeted therapy agent, Lapatinib (GSE16179) (**Figure 5C**)^42^. Next we generated the SS (Delta (r)) by calculating the difference of two Pearson r values for each sample (i.e.: CS/M (r) - NS/E (r)), where negative values indicate a more NS/E phenotype. Using SSs we found that tumor tissues from 29 breast cancer patients obtained before Anthracycline and Taxane based neo-adjuvant chemotherapy were more NS/E-like, compared to post-therapy tumor tissues from 32 patients (GSE28844)^43^, suggesting that chemotherapy exposure had resulted in a general phenotype switch in most of these tumors (**Figure 5D**). Therefore, just like the hierarchical clustering based experiments summarized in **Figure 4**, the SS based approach could identify cells and tumor tissues which responded to chemotherapy treatment. Based on these results, we conclude that CNCL can trace tumor phenotype plasticity.

### Utilization and *in vitro* validation of CNCL as a drug sensitivity predictor

To identify drugs which could preferentially target CS/M and NS/E cells, we analyzed the CCLE database and found 4 drugs that affect these subtypes differentially: Midostaurin and Elesclomol were significantly more effective on CS/M cells, whereas Lapatinib and Panabinostat were cytotoxic for NS/E cells (**Figure 5E**). To validate these *in silico* findings *in vitro*, we first tested whether the CNCL based classifications could be reproduced using qPCR generated data. Indeed, qPCR data obtained for genes in the list were highly concordant with CCLE (GSE36139) microarray expression data (**Supplementary Table 9A**), and inter-gene correlations were as expected (**Supplementary Table 9B**). We then generated a SS matrix based on qPCR data, similar to that generated for *in silico* analyses: BC cell lines were hierarchically clustered using standardized qPCR expression data thus generating CS/M and NS/E cell groups. Each group was used independently to calculate average expression values for all 15 genes which were then used to generate the CS/M and NS/E matrices and ultimately SSs (delta (r) values).

**Figure 6:**
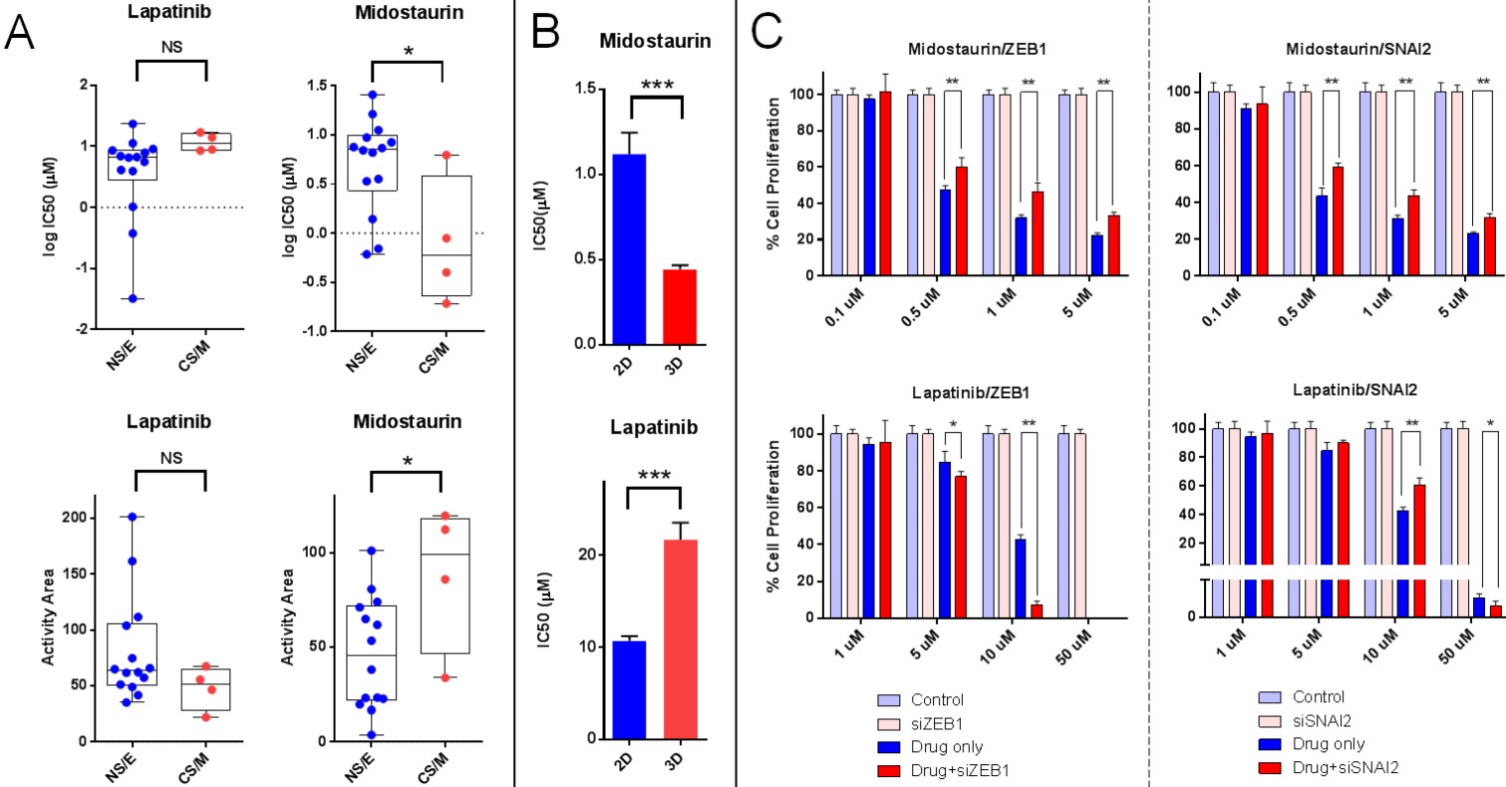
***In vitro* validation of CNCL as a drug sensitivity predictor. A**: Midostaurin is preferentially cytotoxic for CS/M cells compared to NS/E cells, while the reverse is observed for Lapatinib (CyQUANT assay). **B**: MDA-MB-157 spheroids showed increased sensitivity to Midostaurin and resistance to Lapatinib. **C**: ZEB1 and SNAI2 knock down results in decreased sensitivity to Midostaurin and increased sensitivity to Lapatinib in MDA-MB-157 cells. *p<0.05 (t-test), **p<0.01, ***p<0.001, NS: not significant.

*In vitro*, Midostaurin was preferentially cytotoxic for CS/M cells in line with our *in silico* findings. We observed that Lapatinib was more effective on NS/E cells (**Figure 6A** and**Supplementary Figure 3**). We were unable to show a differential effect for Elesclomol and Panobinostat on CS/M vs. NS/E cells *in vitro* (data not shown).

To test if the CNCL signature reflected tumor phenotype plasticity *in vitro* and whether this would relate to a change in drug sensitivity, we generated spheroids from MDA-MD-157 cells and measured gene expression by qPCR, and the simultaneous change in drug sensitivity. The qPCR based initial SS for MDA-MD-157 cells (-0.145) increased to +0.145 upon spheroid formation, reflecting a shift towards a CS/M phenotype. This happened concurrently with a significant increase in sensitivity to Midostaurin and a decrease in sensitivity to Lapatinib (**Figure 6B**). Therefore, spheroid generation from MDA-MD-157 cells caused a change in phenotype that could be quantitated by the CNCL, concurrent with a change in drug sensitivity *in vitro*.

As ZEB1 and SNAI2 are considered critical genes in maintaining a mesenchymal/CSC-like phenotype, we knocked down both genes individually in MDA-MD-157 (**Supplementary Figure 4**) and determined changes in CNCL gene expression and drug sensitivity. SS Delta (r) values changed from +5.1 in control siRNA transfected cells, to -0.12 and -0.01 in ZEB1 and SNAI2 siRNA transfected cells, respectively, indicating a switch from a CS/M to a NS/E phenotype. In parallel to this change in phenotype, we observed significantly increased sensitivity to Lapatinib and decreased sensitivity to Midostaurin (**Figure 6C**). The fact that the change in sensitivity to Lapatinib was more obvious for ZEB1 knock-down cells is likely due to the fact that these underwent a stronger shift towards a NS/E phenotype, compared to SNAI2 knock-down cells.

Cumulatively, these results indicate that tumor cells that are innately of a CS/M phenotype, or those that are induced to become so, are less sensitive to Lapatinib and more sensitive to Midostaurin, compared to cells that are innately NS/E or are induced to become so, which show the opposite pattern of sensitivity.

### Evaluation of CNCL as a prognostic tool for breast cancer

**Figure 7:**
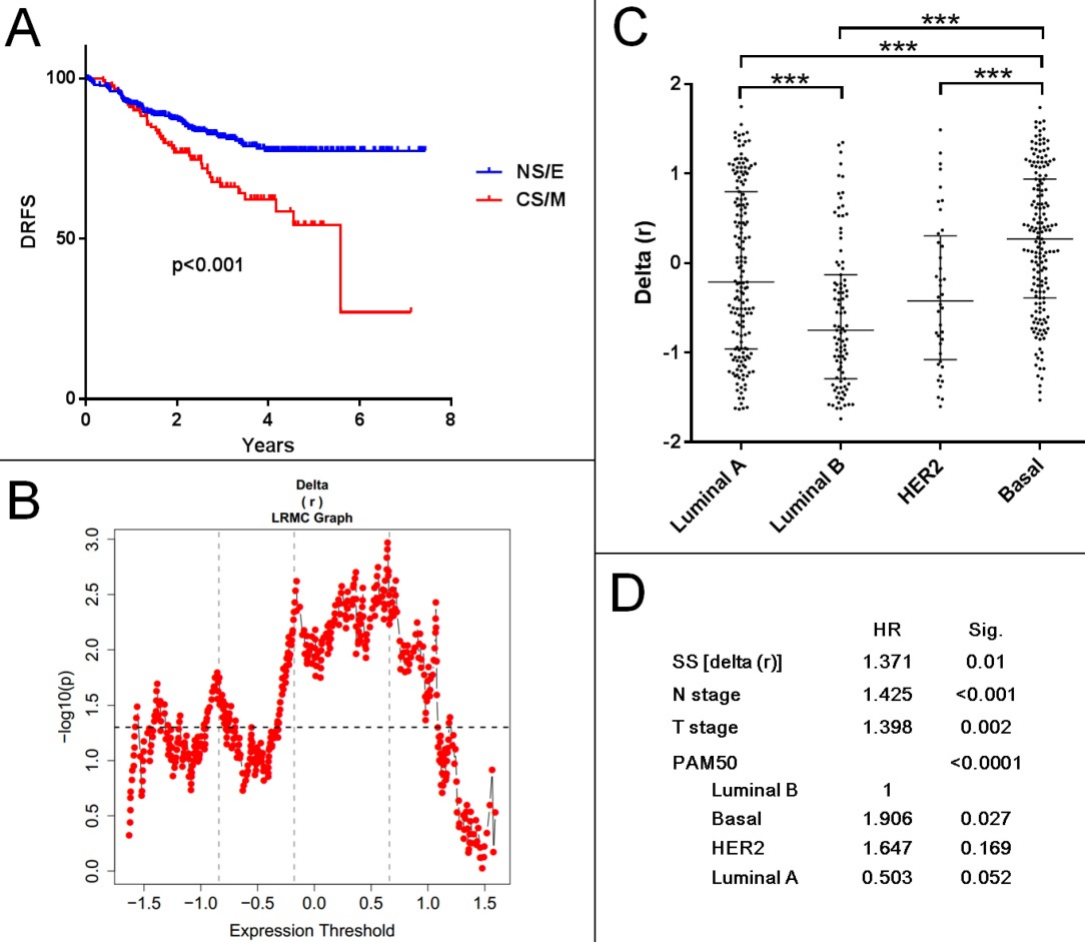
** CNCL can predict prognosis for patients treated with Taxane based neo-adjuvant therapy. A**: (GSE25066) Patients with CS/M tumors show worse prognosis compared to NS/E group, when a SS cut-off of 0.6459 is used. **B**: Log rank test with multiple cut-offs (LRMC). **C**: Delta (r) value distribution among breast cancer subtypes. **D**: Multivariate analysis with multiple clinical parameters showing SS is an independent prognostic factor for patients with paclitaxel neo-adjuvant treatment. ***p<0.001.

To determine if a CNCL based classification of tumor samples could be used to predict prognosis, we generated SS values for all samples within several breast cancer datasets: Metabric cohorts (British (n=994) and Canadian (n=997))^44^, GSE1456 (n=159)^45^, GSE2034 (n=286)^46^, GSE2603 (n=82)^47^, GSE3494 (n=251)^48^, GSE4922(n=249)^49^, GSE6532 (n=380)^50^, GSE7390 (n=198)^51^, GSE11121(n=200)^52^, GSE12276 (n=204)^53^, GSE19615 (n=115)^54^, GSE20685(n=327)^55^, GSE21653 (n=266)^56^, GSE25066(n=508)^57^ and GSE58812 (n=107)^58^. We included all cohorts with survival data and did not use filtering criteria as our aim was to discover any prognostic association the CNCL had, independent of confounding factors. As shown in **Supplementary Table 10**, SS delta (r) based evaluation of prognosis of 15 patient cohorts in 29 tests with various end-point measures revealed statistically significant differences between CS/M and NS/E groups in only 7 tests. And in only two of these (GSE25066 and GSE2603) patients with primarily CS/M tumors had worse prognosis compared to NS/E tumor-harboring patients. Interestingly the GSE25066 cohort, where this trend was most obvious, consisted of patients who had received Taxane (incl. Paclitaxel) based neo-adjuvant therapy. Patients in this cohort with NS/E tumors had longer distant relapse free survival compared to those with CS/M tumors (**Figure 7A, B**). Although SS distributions were significantly different for PAM50 subtypes in this cohort (**Figure 7C**), the CNCL based cut-off was a statistically significant prognostic factor in a multivariate analysis that included N stage, T stage and PAM50 subtypes (**Figure 7D**). In line with our results summarized in **Figure 5**, these show a clear relation between Paclitaxel sensitivity and CNCL identified groups, results obtained for this cohort suggest that the CNCL list might be especially beneficial in determining outcome in patients receiving Taxene based treatment as neo-adjuvant therapy. In 22 tests, we did not observe a significant association between SSs and survival end-measures, and in 5 tests we observed the opposite of what we expected, a direct relation between better prognosis and the CS/M phenotype. Given the very consistent results we obtained for the experiments summarized in the previous sections, we think that the lack of a clear relation between CS/M and worse prognosis in patient cohorts is due to the heterogeneity of tumors, and also because information related to chemotherapy or other treatment was not available for most samples.

## Discussion

There exists a large array of markers that have been reported to identify different subtypes of breast cancer cells^59^. We find that CNCL correlates with CD24 and CD44 expression but not with ALDH. EMT markers for breast cancer seem to be shared to a greater extent among cancers and can be used to classify tumors across tissue-types^60^. The CNCL gene list defined in this study is related to a previously defined EMT gene list^60^ as indicated by the high concordance between the two (data not shown). Similar to the EMT gene list, CNCL can classify both primary tumor tissues as well as cell lines.

The differentiation state of a tumor is accepted as the key determinant of resistance to therapy^61^. However, tumors have the capability of altering their phenotype, which is referred to as phenotype plasticity. This term is used to define both de-differentiation as well as trans-differentiation in cancer cells^62^. Our findings show that phenotype plasticity occurs almost without exception in tumor cells, *in vivo* and *in vitro*, in response to chemotherapy and CNCL is able to dynamically follow the phenotype switch between these two states. As both Zeb1 and Snail1 induction have been associated with EMT and drug resistance^63^, and as Zeb1 is also a critical component of tumor cell plasticity^64^, it is not surprising that the down-regulation of these would correlate with increased sensitivity to anti-cancer agents. Our results clearly show that the CNCL can identify tumor states and cell lines undergone through EMT or stemness as a result of phenotype plasticity. CNCL is therefore, a novel gene list that can account for both stemness and EMT in breast cancer, indicating that the two are related when analyzed in the context of this signature.

Metastasis is considered to be the major reason for tumor related mortality. Although EMT clearly has a role in tumor progression and metastasis, recent data suggests that the metastatic potential of tumor cells and their ability to undergo EMT are not necessarily related^65^. These studies suggest that instead of classifying tumors either as epithelial or mesenchymal, to determine the rate at which plasticity occurs might be a better indicator of prognosis and drug responses^62^. It is therefore, important to be able to quantify the exact phenotypic status of a tumor within the EMT scale and to be able to trace its change. In this line, we believe the CNCL defined here is worth validating in larger studies.

Gene signatures in BC have been shown to be able to predict sensitivity to various drugs *in vitro*^66^ and EMT has been reported to result in resistance to the EGFR inhibitor Gefitinib^67^. Our results suggest the best strategy for BC treatment is possibly the utilization of two drugs simultaneously, each which would target either NS/E or CS/M cells. In line with our earlier findings^27^, we show here for the first time *in vitro*, that Lapatinib and Midostaurin are candidates for such combination therapy, as they target selectively either NS/E or CS/M cells, respectively. Even though EMT might be a signature shared among tumor types, combination treatment suggestions obtained for breast cancer in this study are different from those we found for other tumor types, such as colon cancer and melanoma^68^,^69^. One explanation for this can be that NS/E and CS/M cell types defined for tumors originating from different tissues actually define different phenotypes in the EMT or plasticity scale. Indeed most CS/M cells of colon tumors are similar to NS/E cells of breast cancer, while melanoma cells are among the most CS/M cells when compared to all other tumors^60^.

Our results clearly show that most tumors with a pre-therapy NS/E phenotype will switch to a CS/M phenotype post-therapy. This might explain why most tumors are resistant to secondary treatment. Changes in gene expression following NACT have shown that highly proliferative tumors are more sensitive to chemotherapy^70^,^71^. Here we show that the CNCL gene list can also robustly distinguish patients who will have a better prognosis post taxene-based NACT. However, in light of our findings summarized here and those of others^72^, it should be noted that NACT might induce a CS/M phenotype in tumors, reducing drug responsiveness and thus, overall or disease-free survival. Therefore, it is critical to conclusively determine if NACT is actually decreasing survival of patients whose tumors switch to a CS/M state, following therapy, rather than improving survival. The CNCL could be utilized to follow tumor plasticity in response to such therapies to evaluate responses to NACT and to help answer such questions.

## Conclusions

We show in this study for the first time that the CNCL gene list, which includes stem-cell and EMT features, reflects tumor plasticity and thus a shift in cytotoxicity profiles, especially in response to Midostaurin and Lapatinib in breast cancer; and that it is applicable both *in silico* and *in vitro*. Although the gene list is not uniformly predictive of prognosis, it can be utilized to identify patients who might benefit from Taxene-based neo-adjuvant therapy.

## Supplementary Material

Supplementary figures and tables.

## Abbreviations

CSC: Cancer stem cells
BC: Breast cancer
PR: Progesterone receptor
ER: Estrogen receptor
EMT: Epithelial to mesenchymal transition
MET: Mesenchymal to epithelial transition
CS/M: Cancer-stem-cell-like and mesenchymal
NS/E: Non cancer stem cell like and epithelial
GSEA: Gene set enrichment analysis
CNCL: CSC/non-CSC gene list
