# Supplementary material for: A Stemness and EMT Based Gene Expression Signature Identifies Phenotypic Plasticity and is A Predictive but Not Prognostic Biomarker for Breast Cancer
Source: J Cancer. 2020 Jan 1;11(4):949–61. doi: 10.7150/jca.34649 (PMC6959010; doi:10.7150/jca.34649)
Supplement: Supplementary file 1 — Supplementary figures and tables. [file jcav11p0949s1.pdf]

Supplementary figures and tables

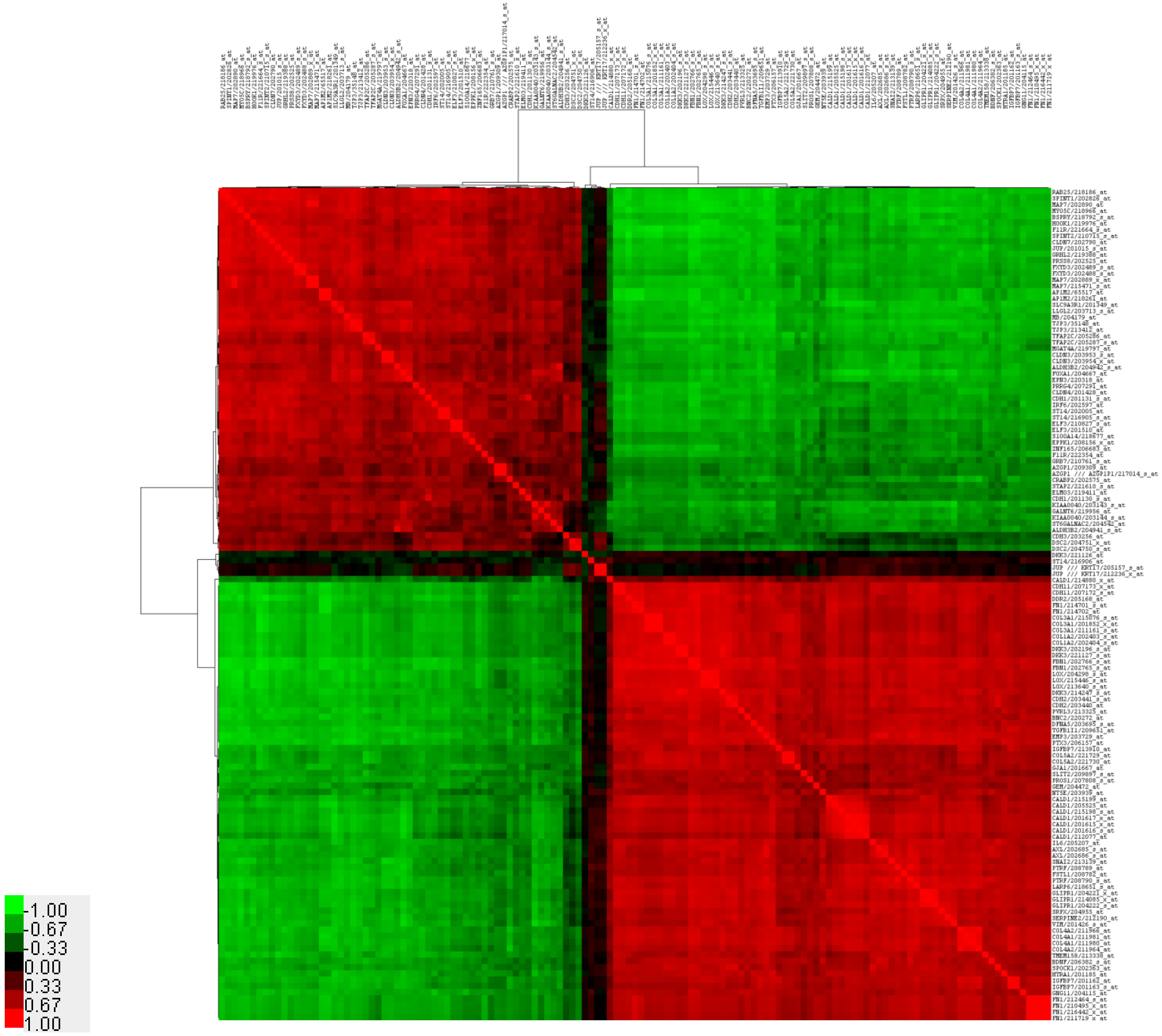

Supplementary Figure 1A. Intergenic Pearson correlation analysis between 133 differentially expressed probesets. Differentially expressed 129 probesets among CSC-like and non-CSC-like cell lines, show strong intergenic relationship through Pearson correlation analysis in GSE36139 (CCLE). Red shows positive correlation and green shows negative correlation. Only 4 probesets: JUP///KRT17 (205157_s_at, 212236_x_at), DKK3 (221126_at) and ST14 (216906_at) show low correlation with other probesets.

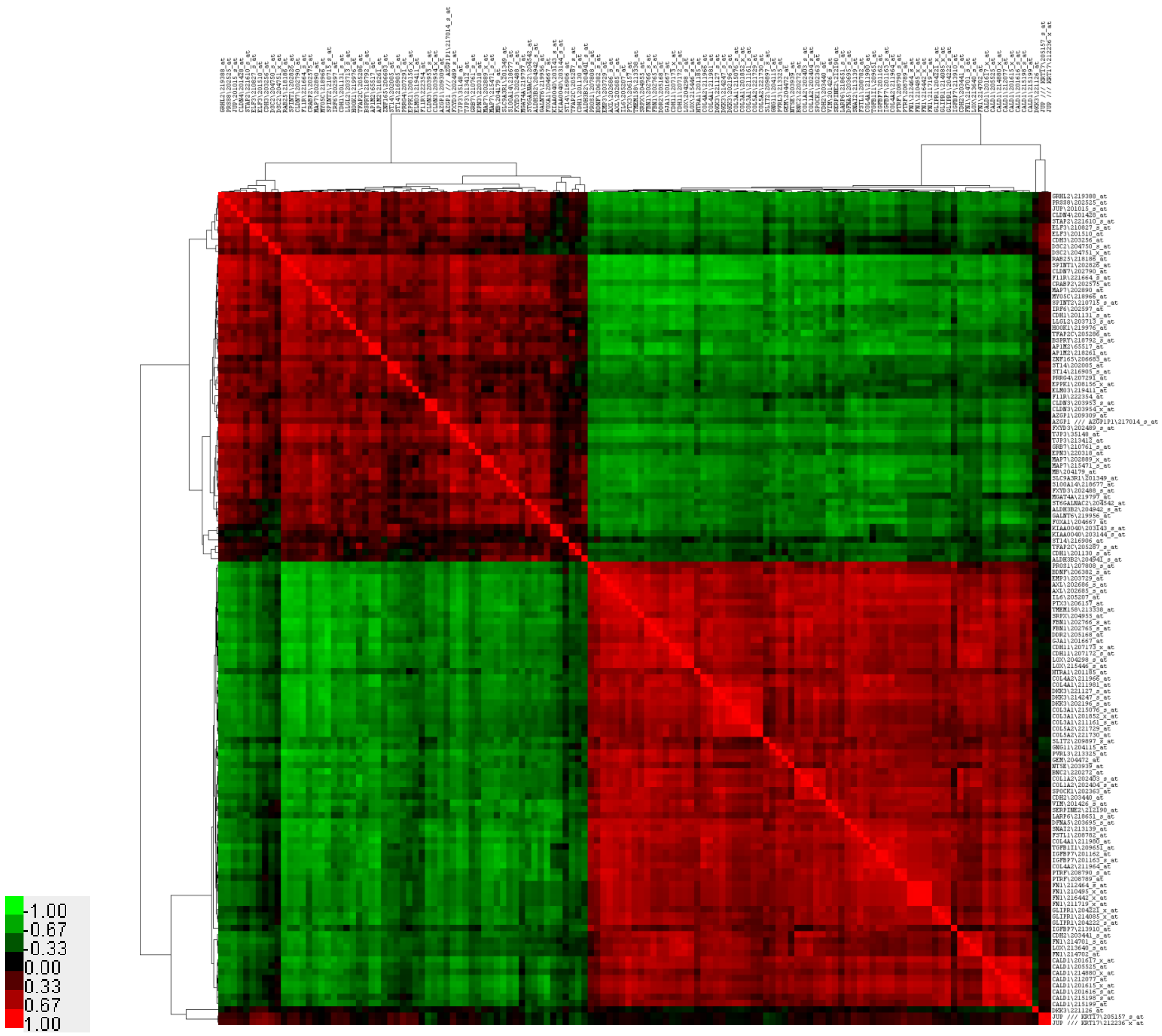

Supplementary Figure 1B. Intergenic Pearson correlation analysis between 133 differentially expressed probesets. Differentially expressed 129 probesets among CSC-like and non-CSC-like cells, show strong intergenic relationship through Pearson correlation analysis in E-MTAB-783 (CGP). Red shows positive correlation and green shows negative correlation. Only 4 probesets: JUP///KRT17 (205157_s_at, 212236_x_at), DKK3 (221126_at) and ST14 (216906_at) show low correlation with other probesets.

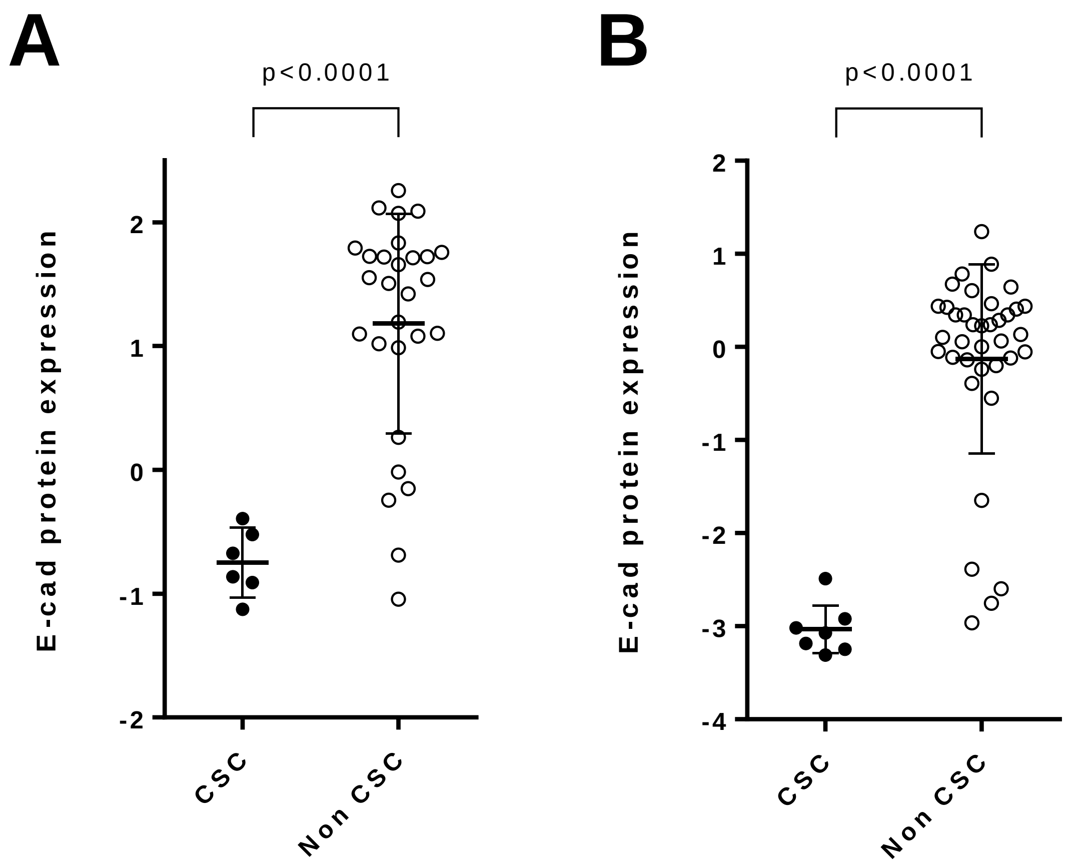

Supplementary Figure 2. Protein array based analysis of E-cad expression. E-cad levels are significantly different in CSC and non-CSC-like cell lines in TCPA (A) and Marcotte et al. (2016) (B) datasets.

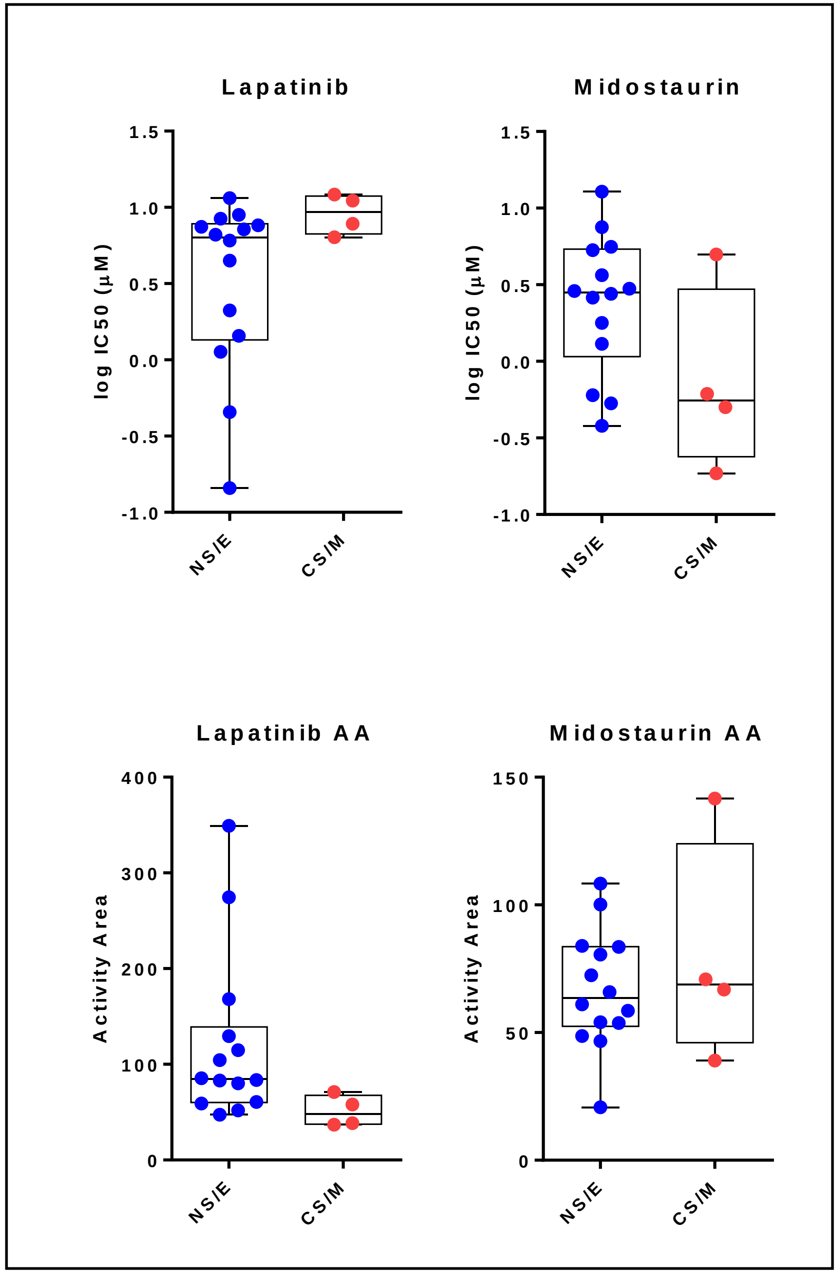

Supplementary Figure 3. Midostaurin shows preferential cytotoxicity for CS/M cells compared to NS/E cells. T-test P values are as follows: Lapatinib IC50: 0.15; Lapatinib AA: 0.14; Midostaurin IC50: 0.08; Midostaurin AA: 0.4 (CellTiter Glo assay)

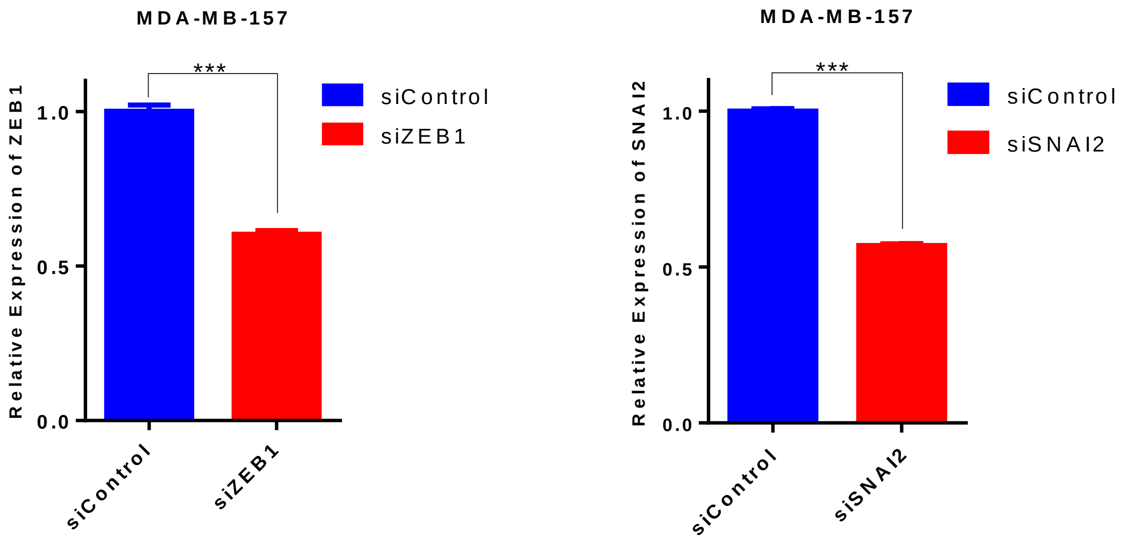

Supplementary Figure 4. Knockdown efficiency of siZEB1 and siSNAI2 in MDA-MB-157. ***: p<0.0001

Supplementary Table 1: Datasets used in this study

Dataset	Original findings	Findings of this study
GSE36139 (CCLE)	Authors developed and analyzed a data resource which contained gene expression, copy number and drug cytotoxicity data for 947 cell lines. They showed that cell lines do represent subtypes of various cancer and drug response data generated here could help in development of personalized therapeutic regimens.	We used 56 breast cancer cell lines' gene expression data to find differentially expressed genes between CS/M and NS/E groups. Drug cytotoxicity data was used to identify drugs which could target discovered groups separately.
E-MTAB-783 (CGP)	Cell lines were screened with 130 different drugs.	We used 39 breast cancer cell lines' gene expression data to find differentially expressed genes between CS/M and NS/E groups. Drug cytotoxicity data was used to identify drugs which could target discovered groups separately.
GSE24717	Authors developed a stemness signature to differentiate between cancer stem cell enriched samples. This signature has prognostic importance and authors recommend to treat stem cell enriched samples with topoisomerase inhibitors and resveratrol.	To identify this signature, authors did not used breast cancer specific cancer stem cell markers (CD44/CD24) but used CD133. But they later showed if they classify stem enriched cell lines from the rest then both markers show same pattern. But on the other hand our signature can identify not only CS/M group (CD44+/CD24-) from NS/E group (CD44-/CD24+) but can also differentiate between resistant and sensitive cell lines to several commercial drugs and also our classification overlaps with epithelial and mesenchymal classification as evidenced in literature as well. We used this dataset to show that using our differentially expressed signature can classify breast cancer cell lines in the same groups which were formed in our discovery dataset, CCLE and CGP.
GSE50811	Authors performed gene expression profiling of breast cancer cell lines and used this data to identify genes which can be related with paclitaxel and eribulin sensitivity. They showed that EMT genes were related eribulin sensitivity.	In this paper authors treated cell lines with paclitaxel and eribulin only for 24 hours before checking their gene expression. This time is not enough for such experiment. So we used only untreated cell line data to further validate our gene signature in clustering breast cancer cell lines. Cell lines were clustered into same groups which were formed in our discovery dataset, CCLE and CGP.
GSE73526	Authors performed shRNA dropout screens on 77 breast cancer cell lines to identify vulnerabilities in breast cancer and associated this data with genomic and proteomic data of those cell lines. Additionally comparing those vulnerabilities with drug data showed potential resistance mechanisms, anticancer effects and need for combination therapies.	We used this dataset to show that using our gene signature can classify breast cancer cell lines in the same groups which were formed via our discovery datasets, CCLE and CGP.
GSE15192	Authors showed that a subpopulation of MCF-10A cells acquire CD44+/CD24- phenotype, and that a few EMT related genes play a role in this switch. They found 2035 genes as differentially expressed, and validated some.	We developed a gene signature that can differentiate between CD44+/CD24- and CD44-/CD24+ phenotypes. And we used gene expression data uploaded by the authors to validate this signature and successfully clustered samples as expected.
GSE36643	Authors investigated a new CSC marker GD2, in HMLER cells and proposed to use this as a single marker of CSC as opposed to CD44 and CD24 markers for breast cancer.	We utilized CD44 and CD24 based distinctions to validate our gene list successfully.
GSE52327	Authors sorted patient derived breast cancer cells based on ALDH, another marker for stemness.	We showed that CD44 and CD24 gene expression does not correlate with ALDH gene expression. We used this dataset to validate this observation.
GSE9691	Authors investigated the role of E-cadherin loss in promoting metastasis and concluded that its loss in breast cancer HMLE cells not only increases their metastatic potential but also increases their invasiveness, motility and resistance to apoptosis.	We could identify E-cad downregulated samples as CS/M from control and beta catenin downregulated samples as NS/E.
GSE24202	Authors used this dataset to associate EMT with breast cancer stem cells. They generated mesenchymal cells HMLE cells by overexpressing TGF beta, Twist, Gsc and by downregulating E-cad. They identified a gene signature of 159 transcription factors responsible for clustering mesenchymal/stem cells from Epithelial/non stem cells.	We used this dataset to successfully distinguish epithelial and mesencymal cell groups generated by the authors with the exception of siE-cad cells, which we explain in figure 3.
GSE7515	Mammosphere culture is associated with enriching cells for cancer stem cells. Authors generated this dataset from human breast tumor cells cultured in adherent conditions and mammosphere culture. Their aim was to identify genes which could distinguish adherent cells from mamamospheres.	We used this datasets to further validate CNCL and most of mamamospheres were clustered as CS/M and primary breast cancer cell lines as NS/E.
GSE24460	Authors generated doxorubicin resistant MCF7 cells which were highly invasive, tumorigenic and formed mamamospheres when compared to control cells. 30% of these MCF7 doxorubicin resistant cells showed CD44+/CD24- phenotype. Genes responsible for drug resistance and stem cell characteristics were high in resistant cells when compared to sensitive cells.	We used this dataset to find if CNCL can identify resistant MCF7 cells from controls. Upon hierarchical clustering, as expected resistant cells were clustered as CS/M separately from control cells as NS/E.
GSE10281	Stem cells are responsible for drug resistance. Authors took biopsies from patients before treatment and after treatment with letrozol for 3 months. They looked at the mesenchymal and epithelial markers and these were differentially expressed in samples before and after letrozol therapy.	We used this dataset to show that CNCL can identify patients before and after undergoing treatment. Half of NS/E samples switched to a CS/M phenotype and only one patient switched in the opposite direction while others maintained their phenotype.
GSE12791	In this dataset, authors developed Paclitaxel resistance in breast cancer cell line MDAMB231 by prolonged drug treatment and studied the effect of bexarotene in switching resistant phenotype back to sensitive.	We used this dataset to successfully cluster Paclitaxel resistant phenotype (CS/M) from sensitive phenotype (NS/E).
GSE23399	Breast cancer associated fibroblasts (CAF) were isolated from patients tumor specimens and were treated with Paclitaxel over a prolonged time. These chemotherapy resistant CAFs are responsible for tumor growth and aggression.	We used this datasets to successfully demonstrate that drug resistant phenotype behaves as CS/M and control cells behave like NS/E cells.
GSE16179	Authors treated breast cancer cell line BT474 with lapatinib over a prolonged period of time and demonstrated that AXL plays a novel role in acquiring resistance to Lapatinib.	We used Lapatinib sensitive and resistant cell models to successfully demonstrate that the resistant phenotype is of a CS/M, while the sensitive phenotype is classified as NS/E.
GSE28844	in this study authors aimed to identify such pathways which confer resistance to tumors post chemotherapy.	We used this dataset to show that tumors treated with Taxane have a higher CS/M score when compared to pre treated samples

Supplementary Table 1: Datasets used in this study (continued)

Survival analysis related datasets	GSE1456	Authors developed a 64 gene signature which can estimate breast cancer patients response to adjuvant therapy.	Our survival analysis using CNCL revealed that patients with NS/E phenotype showed worse prognosis significantly when compared with CS/M phenotype, using disease specific survival, Overall survival and relapse free survival data.
	GSE2034	Authors developed a 76 gene signature which can identify patients at high risk of distant recurrence from patients with favorable prognosis.	CNCL showed no difference in recurrence between CS/M and NS/E patients.
	GSE2603	Authors identified genes which are responsible for breast cancer metastasis to bone and lung tissue.	CNCL showed that patients with CS/M phenotype had worse prognosis when compared with NS/E patients for lymph node metastasis free survival.
	GSE3494	Authors identified a 32 gene signature which can differentiate between p53 wild type and mutant samples, and predicts survival independent of all other prognostic factors.	CNCL showed no significant difference when patients with CS/M phenotype were compared with NS/E patients.
	GSE4922	Authors identified a genetic grade signature which can separate low and high grade disease and can improve therpaeutic decision making for breast cancer patients.	CNCL showed patients with CS/M phenotype showed better prognosis when compared with NS/E patients with border line significance.
	GSE6532	Authors developed a gene grade index which defined histologic grade and found 2 distinct ER+ subgroups with survival difference.	CNCL showed patients with CS/M phenotype showed significantly better prognosis when compared with NS/E patients.
	GSE7390	Authors validated a 76 gene signature for distant metastasis free survival, overall survival, relapse free survival, time to distant metastasis survival.	CNCL showed no survival difference between CS/M and NS/E patients.
	GSE11121	Authors generated and associated several metagenes with distant metastasis free survival (proliferation metagene and B cell metagene)	CNCL showed patients with CS/M phenotype showed better prognosis when compared with NS/E patients which was statistically insignificant.
	GSE12276	Authors identified genes which are responsible for breast cancer metastasis to brain (COX2, HBEGF and ST6GALNAC5).	CNCL showed no survival difference between CS/M and NS/E patients.
	GSE19615	Authors identified 2 genes (LAPTM4B and YWHAZ) as responsible for generation of chemoresistance to anthracyclines.	CNCL showed no survival difference between CS/M and NS/E patients.
	GSE20685	Authors identified molecular subtypes of breast cancer and proposed these subtypes to better customization of breast cancer treatment.	CNCL showed no survival difference between CS/M and NS/E patients.
	GSE21653	Authors suggested ECRG4 as tumor suppressor gene which can be used to better breast cancer prognostication.	CNCL showed no survival difference between CS/M and NS/E patients.
	GSE58812	Authors identified 3 subtypes of triple negative breast cancer and proposed that immune mediation in these tumors can be channeled to treat specific subtypes.	CNCL showed patients with CS/M phenotype showed better prognosis when compared with NS/E patients significantly for metastasis free survival and insignificantly for overall survival.
	GSE25066	Authors developed a genomic predictor for patients treated with taxane and anthracycline chemotherapy.	CNCL showed patients with CS/M phenotype showed worse prognosis when compared with NS/E patients with statistical significance.
	Metabric British Cohort	Authors performed unsupervised analysis of paired DNA RAN profiles and found novel groups with distinct clinical outcomes and then validated these in another cohort.	CNCL showed patients with CS/M phenotype showed worse prognosis when compared with NS/E patients with statistical significance.
	Metabric Canadian Cohort		CNCL showed no survival difference between CS/M and NS/E patients.

Supplementary Table 2: Primer sequences for selected genes

Gene	Primer	Sequence (5'->3')	Length	Tm (°C)
ST14	F	AGAAACCGGCAGAGTACAGC	20	60.04
	R	TTGATGACGCGGATCTCACC	20	60.18
BSPRY	F	CAAGGGTTCTGGCAGTGACT	20	59.89
	R	GGAAGGACACATGATGGGCA	20	60.03
IRF6	F	CGTGCACTATGATGCTTGGC	20	59.97
	R	CCCGACACAGACAGATAGGC	20	59.9
PVRL3	F	GTGGAGCAGGTTGGATGGAC	20	60.68
	R	TGCTAGATCCTCGATGTCAGC	21	59.39
DDR2	F	CTTACCTCCCTCAACCAGCC	20	59.75
	R	GCATGGGTGAGTGGTAGGTC	20	60.11
BNC2	F	TCCTTGACTTGAGCACCACC	20	59.89
	R	ATGATCCCACCATTGCTCCC	20	59.81
ZNF165	F	GGGCTGTCCTACTGATCCTG	20	59.24
	R	GGTTGTCCCCAAGTGTCCTAC	21	60.27
AP1M2	F	GAAACAGTCAGTGGCCAACG	20	59.69
	R	AGTGGGCTCGCATCAAGTAC	20	60.11
SLIT2	F	TGACCAACGGACCAATGACC	20	60.25
	R	CCCATGCTTGCACTTGATCG	20	59.9
DKK3	F	AGTTTCCCCTCTGGCTTGAC	20	59.6
	R	ACTGGTAGAGGCAAAGCAGC	20	60.32
TMEM158	F	ACGTGCCCTAGATTCATGGC	20	60.18
	R	AAATCCTTCCCATGCCCTCC	20	59.74
GAPDH	F	TTCTTTTGCGTCGCCAGCCG	20	61.4
	R	CGACCAAATCCGTTGACTCCGACC	24	66.1
FN1	F	TGTGATCCCGTCGACCAATGCC	22	59.23
	R	TGCCACTCCCCAATGCCACG	20	59.62
VIM	F	CCAAGACACTATTGGCCGCCTGC	23	60.36
	R	GCAGAGAAATCCTGCTCTCCTCGC	24	59.42
CLDN4	F	ACCTGTCCCCGAGAGAGAGTGC	22	59.4
	R	GATTCCAAGCGCTGGGGACGG	21	60.11
E-CAD	F	TGGGCCAGGAAATCACATCCTACA	24	57.57
	R	TTGGCAGTGTCTCTCCAAATCCGA	24	57.8

Supplementary Table 3A: CSC/non-CSC gene list (CNCL): 15 genes were selected as biomarkers to identify CSC like cell lines from Non CSC like cell lines.

Gene Symbol	Gene Name	Probeset ID	Fold change in CCLE	T Test p-value in CCLE	Behavior in CSC like cells
IRF6	Interferon Regulatory Factor 6	202597_at	11.3	1.84E-22	Downregulated
ST14	Suppression Of Tumorigenicity 14	202005_at	14.9	6.88E-26	Downregulated
CDH1	Cadherin 1	201130_s_at	89.9	3.22E-10	Downregulated
BSPRY	B-Box And SPRY Domain Containing protein	218792_s_at	18.4	3.18E-28	Downregulated
CLDN4	Claudin 4	201428_at	27.1	7.63E-14	Downregulated
AP1M2	Adaptor-related Protein Complex 1, mu 2 Subunit	65517_at	10.3	5.81E-07	Downregulated
ZNF165	Zinc finger protein 165 (CT gene)	206683_at	8.3	9.01E-10	Downregulated
PVRL3	Poliovirus Receptor-Related 3	213325_at	20.8	2.68E-15	Upregulated
SLIT2	Slit Homolog 2	209897_s_at	12.4	1.98E-05	Upregulated
BNC2	Basonuclin 2	220272_at	7.6	7.42E-08	Upregulated
DDR2	Discoidin Domain Receptor Tyrosine	205168_at	17.4	6.28E-06	Upregulated
VIM	Vimentin	201426_s_at	129.8	1.68E-18	Upregulated
TMEM158	Transmembrane Protein 158	213338_at	17.9	2.57E-06	Upregulated
FN1	Fibronectin 1	212464_s_at	32.7	1.81E-10	Upregulated
DKK3	Dickkopf WNT Signaling Pathway Inhibitor 3	202196_s_at	21.4	6.45E-05	Upregulated

Supplementary Table 3B: Intergenic Pearson correlation of selected genes in CCLE (top) and CGP (bottom) datasets.

CCLE	FN1	VIM	DDR2	SLIT2	PVRL3	BNC2	TMEM158	DKK3	CLDN4	CDH1	ST14	IRF6	AP1M2	ZNF165	BSPRY
FN1	1	0.682	0.762	0.545	0.645	0.734	0.611	0.684	-0.635	-0.649	-0.68	-0.669	-0.781	-0.537	-0.754
VIM	0.682	1	0.731	0.602	0.75	0.746	0.726	0.644	-0.637	-0.631	-0.705	-0.66	-0.657	-0.612	-0.694
DDR2	0.762	0.731	1	0.673	0.739	0.861	0.742	0.826	-0.78	-0.812	-0.763	-0.754	-0.868	-0.75	-0.767
SLIT2	0.545	0.602	0.673	1	0.71	0.727	0.674	0.622	-0.624	-0.568	-0.586	-0.63	-0.572	-0.558	-0.67
PVRL3	0.645	0.75	0.739	0.71	1	0.724	0.624	0.76	-0.73	-0.687	-0.737	-0.774	-0.683	-0.639	-0.79
BNC2	0.734	0.746	0.861	0.727	0.724	1	0.721	0.775	-0.74	-0.701	-0.721	-0.725	-0.82	-0.639	-0.767
TMEM158	0.611	0.726	0.742	0.674	0.624	0.721	1	0.637	-0.741	-0.713	-0.591	-0.675	-0.616	-0.575	-0.687
DKK3	0.684	0.644	0.826	0.622	0.76	0.775	0.637	1	-0.74	-0.701	-0.666	-0.634	-0.782	-0.673	-0.747
CLDN4	-0.635	-0.637	-0.78	-0.624	-0.73	-0.74	-0.741	-0.74	1	0.733	0.695	0.77	0.693	0.549	0.791
CDH1	-0.649	-0.631	-0.812	-0.568	-0.687	-0.701	-0.713	-0.701	0.733	1	0.751	0.773	0.718	0.637	0.732
ST14	-0.68	-0.705	-0.763	-0.586	-0.737	-0.721	-0.591	-0.666	0.695	0.751	1	0.764	0.726	0.732	0.76
IRF6	-0.669	-0.66	-0.754	-0.63	-0.774	-0.725	-0.675	-0.634	0.77	0.773	0.764	1	0.651	0.658	0.763
AP1M2	-0.781	-0.657	-0.868	-0.572	-0.683	-0.82	-0.616	-0.782	0.693	0.718	0.726	0.651	1	0.659	0.712
ZNF165	-0.537	-0.612	-0.75	-0.558	-0.639	-0.639	-0.575	-0.673	0.549	0.637	0.732	0.658	0.659	1	0.651
BSPRY	-0.754	-0.694	-0.767	-0.67	-0.79	-0.767	-0.687	-0.747	0.791	0.732	0.76	0.763	0.712	0.651	1

CGP	FN1	VIM	DDR2	SLIT2	PVRL3	BNC2	TMEM158	DKK3	CLDN4	CDH1	ST14	IRF6	AP1M2	ZNF165	BSPRY
FN1	1	0.688	0.534	0.436	0.536	0.604	0.643	0.59	-0.448	-0.497	-0.427	-0.471	-0.585	-0.35	-0.602
VIM	0.688	1	0.643	0.648	0.674	0.564	0.703	0.598	-0.379	-0.518	-0.546	-0.57	-0.534	-0.54	-0.39
DDR2	0.534	0.643	1	0.564	0.7	0.821	0.739	0.688	-0.541	-0.596	-0.505	-0.575	-0.767	-0.403	-0.492
SLIT2	0.436	0.648	0.564	1	0.577	0.552	0.513	0.39	-0.253	-0.324	-0.527	-0.49	-0.598	-0.478	-0.427
PVRL3	0.536	0.674	0.7	0.577	1	0.546	0.728	0.524	-0.537	-0.6	-0.631	-0.698	-0.693	-0.511	-0.604
BNC2	0.604	0.564	0.821	0.552	0.546	1	0.763	0.68	-0.497	-0.51	-0.463	-0.534	-0.638	-0.507	-0.513
TMEM158	0.643	0.703	0.739	0.513	0.728	0.763	1	0.55	-0.499	-0.601	-0.432	-0.534	-0.632	-0.52	-0.39
DKK3	0.59	0.598	0.688	0.39	0.524	0.68	0.55	1	-0.515	-0.6	-0.513	-0.565	-0.58	-0.437	-0.476
CLDN4	-0.448	-0.379	-0.541	-0.253	-0.537	-0.497	-0.499	-0.515	1	0.535	0.344	0.381	0.57	0.124	0.518
CDH1	-0.497	-0.518	-0.596	-0.324	-0.6	-0.51	-0.601	-0.6	0.535	1	0.573	0.562	0.556	0.27	0.44
ST14	-0.427	-0.546	-0.505	-0.527	-0.631	-0.463	-0.432	-0.513	0.344	0.573	1	0.576	0.56	0.45	0.452
IRF6	-0.471	-0.57	-0.575	-0.49	-0.698	-0.534	-0.534	-0.565	0.381	0.562	0.576	1	0.522	0.427	0.553
AP1M2	-0.585	-0.534	-0.767	-0.598	-0.693	-0.638	-0.632	-0.58	0.57	0.556	0.56	0.522	1	0.276	0.612
ZNF165	-0.35	-0.54	-0.403	-0.478	-0.511	-0.507	-0.52	-0.437	0.124	0.27	0.45	0.427	0.276	1	0.319
BSPRY	-0.602	-0.39	-0.492	-0.427	-0.604	-0.513	-0.39	-0.476	0.518	0.44	0.452	0.553	0.612	0.319	1

Supplementary Table 4A: Gene sets upregulated in CSC like (CS/M) cell lines.

Selected Upregulated Genesets	CCLE Rank	CGP Rank	Combined Rank
PROTEINACEOUS_EXTRACELLULAR_MATRIX	1	1	2
EXTRACELLULAR_MATRIX	2	2	4
BASEMENT_MEMBRANE	5	5	10
COLLAGEN	7	6	13
METALLOPEPTIDASE_ACTIVITY	12	7	19
BASAL_LAMINA	10	15	25
SKELETAL_DEVELOPMENT	11	17	28
MUSCLE_DEVELOPMENT	9	24	33
CELL_MIGRATION	21	14	35
SULFUR_METABOLIC_PROCESS	31	8	39
TRANSFORMING_GROWTH_FACTOR_BETA_RECEPTOR_SIGNALING_PATHWAY	33	12	45
POSITIVE_REGULATION_OF_RESPONSE_TO_STIMULUS	35	11	46
TRANSMEMBRANE_RECEPTOR_PROTEIN_SERINE_THREONINE_KINASE_SIGNALING_PATHWAY	29	20	49
CELL_CYCLE_ARREST_GO_0007050	34	16	50
VASCULATURE_DEVELOPMENT	18	34	52
AXON_GUIDANCE	22	33	55
REGULATION_OF_CELL_MIGRATION	39	28	67
REGULATION_OF_CELL_GROWTH	49	23	72
METALLOENDOPEPTIDASE_ACTIVITY	30	64	94
REGULATION_OF_G_PROTEIN_COUPLED_RECEPTOR_PROTEIN_SIGNALING_PATHWAY	74	35	109
PEPTIDYL_TYROSINE_MODIFICATION	71	44	115

Supplementary Table 4B: Gene sets upregulated in Non-CSC like (NS/E) cell lines.

Selected Downregulated Gene sets	CCLE Rank	CGP Rank	Combined Rank
TIGHT_JUNCTION	1	1	2
APICAL_JUNCTION_COMPLEX	3	3	6
PROTEIN_BINDING_BRIDGING	10	16	26
POTASSIUM_CHANNEL_ACTIVITY	15	15	30
N_ACETYLTRANSFERASE_ACTIVITY	28	10	38
RESPONSE_TO_HORMONE_STIMULUS	56	7	63
MICROBODY	19	78	97
KINASE_REGULATOR_ACTIVITY	64	65	129
APICOLATERAL_PLASMA_MEMBRANE	2	4	6
CALCIUM_INDEPENDENT_CELL_CELL_ADHESION	4	2	6
INTERCELLULAR_JUNCTION	6	5	11
CELL_JUNCTION	17	13	30
ESTABLISHMENT_AND_OR_MAINTENANCE_OF_CELL_POLARITY	13	20	33
HYDROLASE_ACTIVITY_ACTING_ON_CARBON_NITROGEN_NOT_PEPTIDEBONDSIN_LINEAR_AMIDES	5	37	42
REGULATION_OF_MAPKKK_CASCADE	29	14	43
POTASSIUM_ION_TRANSPORT	24	21	45
N_ACYLTRANSFERASE_ACTIVITY	33	12	45
OXIDOREDUCTASE_ACTIVITY_ACTING_ON_THE_ALDEHYDE_OR_OXO_GROUP_OF_DONORSNAD_OR_NADP_AS_ACCEPTOR	11	41	52
TRANSMEMBRANE_RECEPTOR_PROTEIN_TYROSINE_KINASE_SIGNALING_PATHWAY	25	29	54
RESPONSE_TO_BACTERIUM	50	6	56
OXIDOREDUCTASE_ACTIVITY_ACTING_ON_THE_ALDEHYDE_OR_OXO_GROUP_OF_DONORS	20	51	71

Supplementary Table 5: Consistency of CNCL based CS/M or NS/E assignments for cell lines in five datasets

Cell lines (Datasets in rows)

Datasets	BT549	Hs578T	MDAMB231	CAL51	MDAMB157	CAL120	HCC1395	MDAMB436	HBL100	Hs274T	Hs281T	Hs343T	Hs606T	Hs739T	Hs742T	SKBR7
CCLE	CS/M	CS/M	CS/M	CS/M	CS/M	CS/M	CS/M	CS/M	N/A	CS/M	CS/M	CS/M	CS/M	CS/M	CS/M	N/A
CGP	CS/M	CS/M	CS/M	CS/M	CS/M	CS/M	CS/M	N/A	N/A	N/A	N/A	N/A	N/A	N/A	N/A	N/A
GSE73526	CS/M	CS/M	CS/M	CS/M	CS/M	CS/M	CS/M	CS/M	CS/M	N/A	N/A	N/A	N/A	N/A	N/A	CS/M
GSE50811	CS/M	CS/M	CS/M	CS/M	N/A	N/A	N/A	CS/M	N/A	N/A	N/A	N/A	N/A	N/A	N/A	N/A
GSE24717	CS/M	CS/M	CS/M	N/A	CS/M	N/A	N/A	N/A	N/A	N/A	N/A	N/A	N/A	N/A	N/A	N/A

Datasets	SUM1315	SUM159	BT20	HCC1806	MCF7	T47D	BT474	DU4475	HCC1143	HCC1428	HCC1937	HCC1954	HCC2218	HCC38	HCC70	MDAMB361
CCLE	N/A	N/A	NS/E	NS/E	NS/E	NS/E	NS/E	NS/E	NS/E	NS/E	NS/E	NS/E	NS/E	NS/E	NS/E	NS/E
CGP	N/A	N/A	NS/E	NS/E	NS/E	NS/E	N/A	NS/E	NS/E	N/A	NS/E	NS/E	NS/E	NS/E	NS/E	NS/E
GSE73526	CS/M	CS/M	NS/E	NS/E	NS/E	NS/E	NS/E	NS/E	NS/E	NS/E	NS/E	NS/E	NS/E	NS/E	NS/E	NS/E
GSE50811	N/A	N/A	NS/E	NS/E	NS/E	NS/E	NS/E	NS/E	NS/E	NS/E	NS/E	NS/E	NS/E	NS/E	NS/E	N/A
GSE24717	N/A	N/A	NS/E	NS/E	NS/E	NS/E	NS/E	N/A	N/A	NS/E	N/A	N/A	N/A	N/A	N/A	NS/E

Datasets	MDAMB453	MDAMB468	SKBR3	UACC812	AU565	BT483	CAL148	CAL851	CAMA1	EFM19	HCC1187	HCC1419	HCC1500	HCC1599	MDAMB134VI	MDAMB175VII
CCLE	NS/E	NS/E	NS/E	NS/E	NS/E	NS/E	NS/E	NS/E	NS/E	NS/E	NS/E	NS/E	NS/E	NS/E	NS/E	NS/E
CGP	NS/E	NS/E	N/A	NS/E	N/A	N/A	NS/E	NS/E	NS/E	NS/E	NS/E	NS/E	N/A	NS/E	NS/E	NS/E
GSE73526	NS/E	NS/E	NS/E	NS/E	NS/E	NS/E	NS/E	NS/E	NS/E	NS/E	NS/E	NS/E	NS/E	NS/E	NS/E	NS/E
GSE50811	NS/E	NS/E	NS/E	NS/E	NS/E	N/A	N/A	N/A	N/A	N/A	N/A	CS/M	NS/E	N/A	N/A	N/A
GSE24717	N/A	N/A	NS/E	N/A	N/A	NS/E	N/A	N/A	N/A	N/A	N/A	N/A	N/A	N/A	N/A	N/A

Datasets	MDAMB415	UACC893	ZR7530	EFM192A	EVSAT	HCC1569	HCC202	HCC2157	HDQP1	JIMT1	KPL1	MFM223	OCUBM	ZR751	184A1	184B5
CCLE	NS/E	NS/E	NS/E	NS/E	N/A	NS/E	NS/E	NS/E	NS/E	NS/E	NS/E	N/A	N/A	NS/E	N/A	N/A
CGP	NS/E	N/A	NS/E	N/A	NS/E	N/A	N/A	NS/E	N/A	N/A	N/A	NS/E	NS/E	N/A	N/A	N/A
GSE73526	NS/E	NS/E	NS/E	NS/E	NS/E	NS/E	NS/E	N/A	NS/E	NS/E	NS/E	NS/E	NS/E	NS/E	NS/E	NS/E
GSE50811	N/A	NS/E	N/A	N/A	N/A	N/A	N/A	N/A	N/A	N/A	N/A	N/A	N/A	N/A	N/A	N/A
GSE24717	N/A	N/A	N/A	N/A	N/A	N/A	N/A	N/A	N/A	N/A	N/A	N/A	N/A	N/A	N/A	N/A

Datasets	600MPE	COLO824	HCC1008	HCC2185	HCC2688	HCC3153	HCC712	LY2	MACLS2	MB157	MCF10A	MCF12A	MDAMB330	MRKnu1	MX1	SKBR5
CCLE	N/A	N/A	N/A	N/A	N/A	N/A	N/A	N/A	N/A	N/A	N/A	N/A	N/A	N/A	N/A	N/A
CGP	N/A	NS/E	N/A	N/A	N/A	N/A	N/A	N/A	N/A	N/A	N/A	N/A	N/A	NS/E	N/A	N/A
GSE73526	NS/E	N/A	NS/E	NS/E	NS/E	NS/E	NS/E	NS/E	NS/E	NS/E	NS/E	NS/E	NS/E	N/A	NS/E	NS/E
GSE50811	N/A	N/A	N/A	N/A	N/A	N/A	N/A	N/A	N/A	N/A	N/A	N/A	N/A	N/A	N/A	N/A
GSE24717	N/A	N/A	N/A	N/A	N/A	N/A	N/A	N/A	N/A	N/A	N/A	N/A	N/A	N/A	N/A	N/A

Datasets	SUM102	SUM149	SUM185	SUM190	SUM225	SUM229	SUM44	SUM52	SW527	UACC3199	YMB1	ZR75	ZR75B	ZRT
CCLE	N/A	N/A	N/A	N/A	N/A	N/A	N/A	N/A	N/A	N/A	NS/E	N/A	N/A	N/A
CGP	N/A	N/A	N/A	N/A	N/A	N/A	N/A	N/A	N/A	N/A	N/A	N/A	N/A	N/A
GSE73526	NS/E	NS/E	NS/E	NS/E	NS/E	NS/E	NS/E	NS/E	NS/E	NS/E	N/A	N/A	NS/E	N/A
GSE50811	N/A	N/A	N/A	N/A	N/A	N/A	N/A	N/A	N/A	N/A	N/A	N/A	N/A	NS/E
GSE24717	N/A	N/A	N/A	N/A	N/A	N/A	N/A	N/A	N/A	N/A	N/A	NS/E	N/A	N/A

NA: cell line data not available for that dataset

Supplementary Table 6: CNCL correlation (Pearson) with CD44 and CD24 in CCLE (GSE36139) (A) and CGP (E-MTAB-783) (B)

A

Pearson Correlation			
**Gene symbols**	**CD44**	**CD24**	**ALDH1**
**FN1**	0.57	-0.73	-0.07
**VIM**	0.69	-0.67	-0.04
**DDR2**	0.49	-0.86	-0.06
**SLIT2**	0.47	-0.66	0.08
**PVRL3**	0.5	-0.74	0.07
**BNC2**	0.55	-0.72	-0.03
**TMEM158**	0.61	-0.74	-0.14
**DKK3**	0.45	-0.84	-0.15
**CLDN4**	-0.47	0.73	-0.06
**CDH1**	-0.37	0.78	0.1
**ST14**	-0.45	0.7	0.06
**IRF6**	-0.35	0.7	0.04
**AP1M2**	-0.43	0.73	-0.01
**ZNF165**	-0.27	0.73	0.08
**BSPRY**	-0.53	0.78	-0.12

p value			
**Gene symbols**	**CD44**	**CD24**	**ALDH1**
**FN1**	<0.001	<0.001	0.61
**VIM**	<0.001	<0.001	0.8
**DDR2**	<0.001	<0.001	0.69
**SLIT2**	<0.001	<0.001	0.54
**PVRL3**	<0.001	<0.001	0.59
**BNC2**	<0.001	<0.001	0.82
**TMEM158**	<0.001	<0.001	0.3
**DKK3**	<0.001	<0.001	0.28
**CLDN4**	<0.001	<0.001	0.64
**CDH1**	<0.001	<0.001	0.48
**ST14**	<0.001	<0.001	0.68
**IRF6**	0.01	<0.001	0.79
**AP1M2**	<0.001	<0.001	0.94
**ZNF165**	0.04	0	0.54
**BSPRY**	<0.001	<0.001	0.38

Pearson Correlation with ALDH		
**Gene Symbol**	**Pearson r**	**p value**
**CD44**	0.2	0.2
**CD24**	0.2	0.3

B

Pearson Correlation			
**Gene symbols**	**CD44**	**CD24**	**ALDH1**
**FN1**	0.5	-0.57	-0.06
**VIM**	0.6	-0.56	0.04
**DDR2**	0.38	-0.54	0.15
**SLIT2**	0.21	-0.38	0.33
**PVRL3**	0.35	-0.66	0.31
**BNC2**	0.34	-0.58	-0.01
**TMEM158**	0.44	-0.71	-0.05
**DKK3**	0.37	-0.55	-0.11
**CLDN4**	-0.08	0.48	-0.08
**CDH1**	-0.32	0.69	0.19
**ST14**	-0.33	0.41	-0.05
**IRF6**	-0.23	0.57	-0.11
**AP1M2**	-0.25	0.59	-0.08
**ZNF165**	-0.23	0.33	-0.13
**BSPRY**	-0.13	0.63	-0.22

p value			
**Gene symbols**	**CD44**	**CD24**	**ALDH1**
**FN1**	**<0.001**	**<0.001**	**0.68**
**VIM**	<0.001	<0.001	0.81
**DDR2**	0.01	<0.001	0.35
**SLIT2**	0.17	0.01	0.03
**PVRL3**	0.02	<0.001	0.05
**BNC2**	0.03	<0.001	0.97
**TMEM158**	<0.001	<0.001	0.73
**DKK3**	0.02	<0.001	0.51
**CLDN4**	0.6	<0.001	0.61
**CDH1**	0.04	<0.001	0.23
**ST14**	0.03	0.01	0.74
**IRF6**	0.14	<0.001	0.48
**AP1M2**	0.11	<0.001	0.6
**ZNF165**	0.15	0.03	0.41
**BSPRY**	0.41	<0.001	0.17

Pearson Correlation with ALDH		
**Gene Symbol**	**Pearson r**	**p value**
**CD44**	<0.001	0.99
**CD24**	0.08	0.6

Supplementary Table 7: CNCL correlation (Pearson) with CD44 and CD24 in GSE15192 (A) and in GSE36643 (B)

A

Pearson Correlation			
**Gene symbols**	**CD44**	**CD24**	**ALDH1**
**FN1**	0.72	-1	-0.65
**VIM**	0.69	-1	-0.61
**DDR2**	-0.2	0.38	-0.03
**SLIT2**	0.67	-1	-0.72
**PVRL3**	0.68	-1	-0.61
**BNC2**	0.64	-0.9	-0.75
**TMEM158**	0.71	-1	-0.54
**DKK3**	0.36	-0.8	-0.43
**CLDN4**	-0.8	1	0.61
**CDH1**	-0.7	1	0.62
**ST14**	-0.7	1	0.59
**IRF6**	-0.7	1	0.61
**AP1M2**	-0.7	0.99	0.62
**ZNF165**	-0.7	0.99	0.59
**BSPRY**	-0.6	0.99	0.62

p value			
**Gene symbols**	**CD44**	**CD24**	**ALDH1**
**FN1**	0.04	<0.001	0.08
**VIM**	0.06	<0.001	0.11
**DDR2**	0.59	0.36	0.94
**SLIT2**	0.07	<0.001	0.04
**PVRL3**	0.06	<0.001	0.11
**BNC2**	0.09	<0.001	0.03
**TMEM158**	0.05	<0.001	0.17
**DKK3**	0.38	0.03	0.29
**CLDN4**	0.03	<0.001	0.11
**CDH1**	0.06	<0.001	0.1
**ST14**	0.05	<0.001	0.13
**IRF6**	0.07	<0.001	0.11
**AP1M2**	0.08	<0.001	0.1
**ZNF165**	0.08	<0.001	0.13
**BSPRY**	0.1	<0.001	0.1

B

Pearson Correlation			
**Gene symbols**	**CD44**	**CD24**	**ALDH1**
**FN1**	-0.91	-0.88	-0.77
**VIM**	-0.9	-0.99	-0.69
**DDR2**	-0.99	-0.94	-0.93
**SLIT2**	-0.88	-0.85	-0.73
**PVRL3**	-0.95	-0.94	-0.81
**BNC2**	0.09	-0.25	0.25
**TMEM158**	-0.85	-0.92	-0.62
**DKK3**	-0.74	-0.93	-0.47
**CLDN4**	0.9	0.99	0.73
**CDH1**	0.97	0.98	0.84
**ST14**	0.95	0.95	0.8
**IRF6**	0.9	0.96	0.69
**AP1M2**	0.97	0.94	0.84
**ZNF165**	0.96	0.88	0.86
**BSPRY**	0.84	0.97	0.63

p value			
**Gene symbols**	**CD44**	**CD24**	**ALDH1**
**FN1**	0.01	0.02	0.07
**VIM**	0.02	<0.001	0.13
**DDR2**	<0.001	0.01	0.01
**SLIT2**	0.02	0.03	0.1
**PVRL3**	<0.001	0.01	0.05
**BNC2**	0.87	0.63	0.63
**TMEM158**	0.03	0.01	0.19
**DKK3**	0.1	0.01	0.34
**CLDN4**	0.02	<0.001	0.1
**CDH1**	<0.001	<0.001	0.04
**ST14**	<0.001	<0.001	0.06
**IRF6**	0.02	<0.001	0.13
**AP1M2**	<0.001	<0.001	0.04
**ZNF165**	<0.001	0.02	0.03
**BSPRY**	0.03	<0.001	0.18

Supplementary Table 8: CNCL Stemness Matrix

Gene symbol/Probeset ID	Gene related to	CS/M matrix*	NS/E matrix**
DDR2\U\205168_at	CS/M	1.87	-0.507
DKK3\U\202196_s_at	CS/M	1.866	-0.524
SLIT2\U\209897_s_at	CS/M	1.698	-0.588
PVRL3\U\213325_at	CS/M	1.601	-0.7
BNC2\U\220272_at	CS/M	1.544	-0.662
TMEM158\U\213338_at	CS/M	1.534	-0.689
FN1\U\212464_s_at	CS/M	1.498	-0.312
VIM\U\201426_s_at	CS/M	1.351	-0.815
ST14\D\202005_at	NS/E	-1.447	0.544
IRF6\D\202597_at	NS/E	-1.496	0.509
BSPRY\D\218792_s_at	NS/E	-1.539	0.625
ZNF165\D\206683_at	NS/E	-1.566	0.537
CLDN4\D\201428_at	NS/E	-1.572	0.515
CDH1\D\201131_s_at	NS/E	-1.672	0.717
AP1M2\D\65517_at	NS/E	-1.807	0.518

*Mean standardized expression values in CS/M cells in CCLE

**Mean standardized expression values in NS/E cells in CCLE

Supplementary Table 9A: qPCR expression data is concordant with CCLE (GSE36139) microarray data.

Gene	Pearson r	p
VIM	0.94	<0.0001
ST14	0.93	<0.0001
CDH1	0.93	<0.0001
AP1M2	0.92	<0.0001
IRF6	0.91	<0.0001
BSPRY	0.89	<0.0001
DKK3	0.81	<0.0001
PVRL3	0.8	<0.0001
BNC2	0.78	0.0002
FN1	0.72	<0.001
TMEM158	0.7	0.002
DDR2	0.69	0.002
SLIT2	0.69	0.002
ZNF165	0.64	<0.01
CLDN4	0.46	0.06

Supplementary Table 9B: Reproduction of intergenic correlations by qPCR expression data

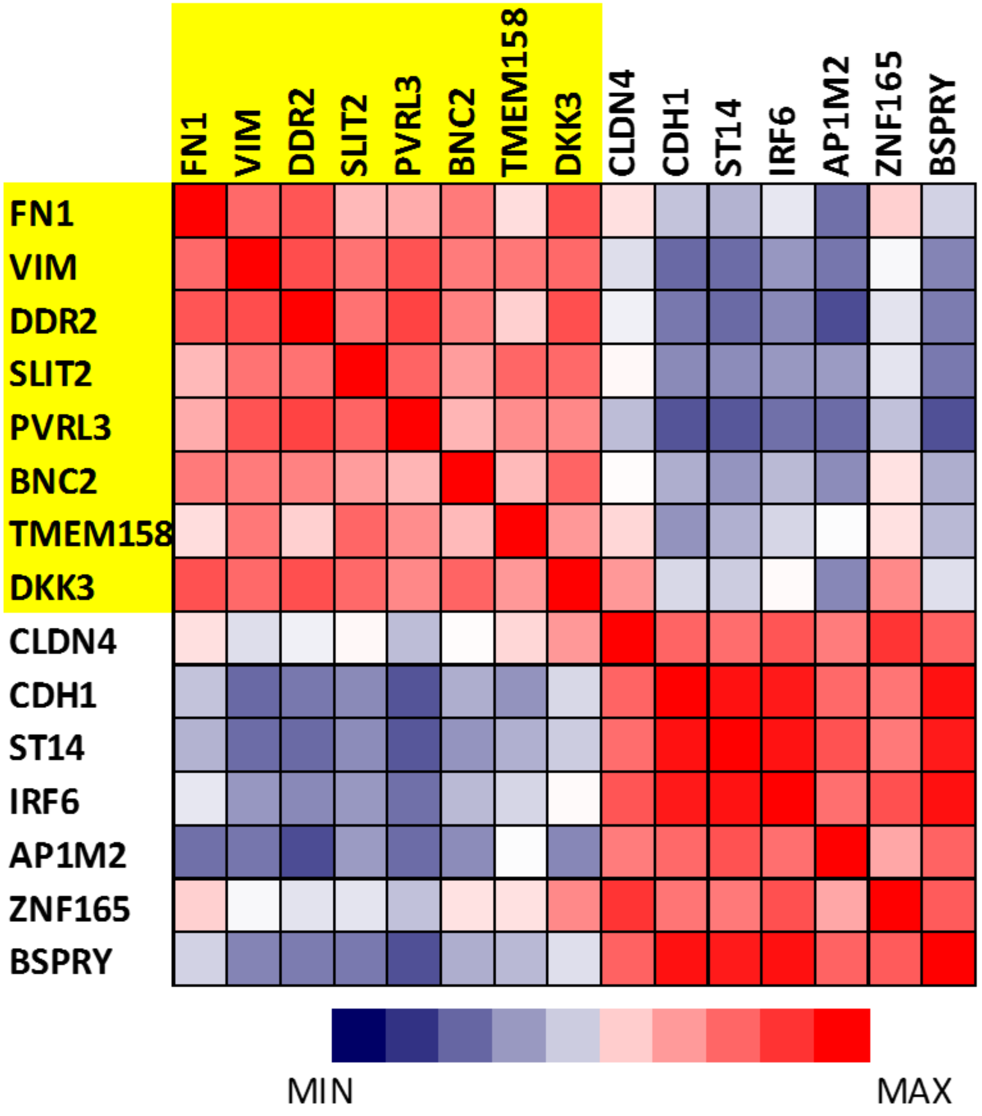

Supplementary Table 10: SS based evaluation of prognosis in 16 breast cancer cohorts.

Dataset	End point measure	Patient numbers				Median survival		Overall Median Survival	Hazard ratio	Cox p value	Summary of treatment protocol
		CS/M	CS/M (censored)	NS/E	NS/E (censored)	CS/M	NS/E				
GSE1456	DSS	129	88%	30	57%	NA	NA	NA	0.524	**0.004**	Adjuvant tamoxifen therapy
	OS	132	81%	27	44%	NA	NA	NA	0.557	**0.002**	
	RFS	129	81%	30	50%	NA	NA	NA	0.579	**0.004**	
GSE2034	BR	57	93%	229	97%	NA	NA	NA	1.263	0.52	No neoadjuvant or adjuvant therapy
	Relapse	143	59%	143	66%	NA	NA	NA	1.073	0.53	
GSE2603	MFS	30	50%	52	77%	6.8	NA	NA	1.426	0.14	ND
	LNMFS	23	65%	59	90%	NA	NA	NA	**2.017**	**0.03**	
	BMFS	24	71%	58	88%	NA	NA	NA	1.569	0.17	
GSE3494	DSS	57	86%	194	68%	NA	NA	NA	0.802	0.08	Adjuvant chemotherapy
GSE4922	OS	203	68%	46	48%	NA	NA	NA	0.808	0.059	Adjuvant tamoxifen therapy
GSE6532	DMFS	64	89%	316	72%	12.6	12.5	12.6	0.754	**0.011**	ND
GSE7390	DMFS	163	67%	35	74%	18.6	NA	19.7	0.985	0.92	No therapy
	OS	58	76%	140	70%	NA	NA	NA	0.968	0.83	ND
	RFS	58	76%	140	70%	18.1	13.5	16	0.968	0.83	ND
	TDM	55	80%	143	72%	19.7	NA	19.7	0.915	0.57	ND
GSE11121	DMFS	69	88%	131	71%	NA	NA	NA	0.763	0.08	Only surgery
GSE12276	RFS	21	10%	183	9%	1.9	1.8	1.8	0.981	0.81	Ajuvant therapy
	BR	21	52%	183	45%	2.8	2.8	2.8	0.971	0.77	
	Brain relapse	21	95%	183	92%	NA	NA	NA	0.941	0.82	
	Lung relapse	158	75%	46	87%	NA	5	NA	1.045	0.78	
GSE19615	RFS	60	90%	55	84%	NA	NA	NA	1.021	0.94	Adjuvant anthracycline therapy
GSE20685	DSS	271	73%	56	84%	14	NA	14.1	1.023	0.84	Adjuvant chemotherapy
	MFS	292	73%	35	86%	NA	NA	NA	1.013	0.91	
GSE21653	DFS	126	67%	140	61%	3.8	NA	4	0.886	0.33	Adjuvant chemotherapy
GSE58812	MFS	67	79%	40	58%	NA	NA	NA	0.642	**0.043**	Adjuvant chemotherapy
	OS	95	76%	12	50%	NA	NA	NA	0.76	0.21	
GSE25066	DRFS	149	70%	359	81%	NA	NA	NA	**1.297**	**0.011**	Neoadjuvant paclitaxel therapy
Metabric Canadian Cohort	OS	120	72%	874	52%	12.2	12.4	12.4	0.965	0.5	Hormonal, radio- and chemotherapy
Metabric British Cohort	OS	402	61%	595	51%	13.2	10.8	12.2	0.895	**0.03**	Hormonal, radio- and chemotherapy

DSS: disease specific survival, OS: Overall survival, RFS: Relapse free survival, BR: Bone relapse, MFS: metastasis free survival, LNMFS: Lymph node metastasis free survival, BMFS: Bone metastasis free survival, DMFS: distant metastasis free survival, DRFS: Distant relapse free survival, DFS: disease free survival, TDM: time to distant metastasis, ND: not disclosed
